# Quantum‐Inspired Fourier Transforms Based on Circuits

**DOI:** 10.1002/advs.202510261

**Published:** 2025-08-21

**Authors:** Hanxu Zhang, Yifan Sun, Xiangdong Zhang

**Affiliations:** ^1^ Key Laboratory of advanced optoelectronic quantum architecture and measurements of Ministry of Education Beijing Key Laboratory of Nanophotonics & Ultrafine Optoelectronic Systems School of Physics Beijing Institute of Technology Beijing 100081 China

**Keywords:** circuit gates, classical circuit networks, Fourier transform, quantum algorithm, signal processing

## Abstract

Fourier transform (FT) is ubiquitous in modern society due to their broad applications in many branches of science and engineering. Improving the speed of FT is a common interest in the fields of signal processing. The quantum FT is generally believed to be superior to classical algorithms, but it requires a special quantum environment to perform, which has not yet been widely used. Inspired by quantum FT, here a new FT scheme is demonstrated based on circuits. In the circuit scheme, a new type of classical correlation, which its mathematical form corresponds to those of quantum entanglement, has been constructed. The calculation speed using the designed circuit scheme is equivalent to those based on the quantum FT algorithms, which is faster than those based on the classical fast FT algorithms. Furthermore, some basic gates have been designed and experimentally fabricated using classical circuit networks, which can emulate the functions of quantum gates. Fast calculation efficiencies for the FT based on the designed classical circuit networks have been demonstrated. Extensive applications of the FT scheme in the signal processing are anticipated.

## Graphical abstract

An analog scheme of classical circuit for the quantum Fourier transform is proposed. The number of the basic computing components employed in the circuit design is consistent with the number of the quantum gate in the quantum circuit. The information is encoded using correlated electrical signals. This makes the classical circuit network have processing functions similar to that of quantum computation. Furthermore, an experimental verification on the analogy of the two, three and five qubit processors is presented.

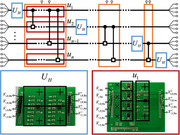

## 1 Introduction

Fourier transform (FT) becomes a standard tool in contemporary sciences since the ‘fast Fourier transform (FFT)’ algorithm was developed in 1965.^[^ ^1^ ^]^ It has been used in many fields such as physics, number calculation, signal processing, probability statistics, cryptography, acoustics, and optics.^[^ ^2^, ^3^ ^]^ The FFT plays an important role in these fields. On the other hand, with the progress of research in quantum information, the quantum FT algorithm has been proposed.^[^ ^4^, ^5^, ^6^, ^7^, ^8^, ^9^ ^]^ The quantum FT (QFT) is believed to outperform the classical FFT in signal processing, which has attracted much attention in recent decades.^[^ ^10^, ^11^, ^12^, ^13^, ^14^, ^15^, ^16^, ^17^, ^18^, ^19^ ^]^ However, the QFT is typically believed to be performed in a quantum computing environment, where information is encoded using quantum states. Because quantum states are quite fragile and easily affected by external disturbance, the bottleneck issues such as decoherence and scalability are particularly difficult to address.^[^ ^9^, ^20^ ^]^ Although there have been significant progresses in the research of quantum information process in recent years,^[^ ^21^, ^22^, ^23^, ^24^, ^25^, ^26^, ^27^, ^28^, ^29^, ^30^, ^31^ ^]^ applying QFT to solve practical problems still keeps great challenges.

Recent investigations have shown that a formal analogy between classical and quantum information processes can be constructed.^[^ ^32^, ^33^, ^34^, ^35^, ^36^, ^37^, ^38^, ^39^, ^40^, ^41^, ^42^, ^43^, ^44^, ^45^, ^46^, ^47^, ^48^, ^49^, ^50^, ^51^, ^52^, ^53^ ^]^ Based on such a correspondence, the QFT has been emulated in some classical systems.^[^ ^54^, ^55^, ^56^ ^]^ For example, an electric circuit scheme for performing the QFT has been discussed in a recent work, by using the simulation of quantum walks.^[^ ^56^ ^]^ However, the proposed scheme is not scalable. Namely, it needs 2*^n^* number of circuit components to realize *n* qubits. This means that the scheme failed to provide a classical analogy of QFT circuit in sources. The question is whether it is possible to design classical circuits to implement better analogy of QFT circuit.

Inspired by the QFT circuit scheme, in this work we propose an analog scheme of classical circuit for the QFT. In our scheme, the classical circuit network, which includes the information processing elements that corresponds to the quantum gate sets, is designed. The information is encoded using correlated electrical signals, which establishes a mathematical correspondence between the novel classical correlation and quantum entanglement. This makes the classical circuit network have processing functions similar to that of quantum computation. As a results, the number of the basic computing components employed in our circuit, including only the copy, addition, subtraction and multiplication operations, is consistent with the number of the quantum gate in quantum circuit. Based on our theoretical consideration, we present an experimental verification on the analogy of the two, three and five qubit processors.

## 2 Theoretical Scheme of Quantum‐Inspired Fast Fourier Transforms Based on Circuits

The *n*‐qubit QFT is a basis transformation in an *N* = 2^*n*^‐state space that transforms the state |*K*〉 to |*J*〉 (K and J are integers ranging from 0 to *N* − 1) according to

(1) $$|K\rangle \overset{\mathrm{QFT}}{\to }\frac{1}{\sqrt{N}}\sum_{J=0}^{N-1}e^{i2\pi KJ/N}|J\rangle ,$$

(2) $$\begin{array}{c} \sum_{K=1}^{N}x_{K}|K\rangle \overset{\mathrm{QFT}}{\to }\sum_{J=1}^{N}X_{J}|J\rangle \\ \;\;\;\;=\sum_{J=1}^{N}\frac{1}{\sqrt{N}}\sum_{K=1}^{N}x_{k}e^{i2\pi KJ/N}|K\rangle , \end{array}$$

where the *N* data points *x*_1_,*x*_2_,⋅⋅⋅, *x*_*N* − 1_ and *x_N_* are encoded into the *N* amplitudes of the *N* basis states, and the output amplitudes *X*_1_,*X*_2_,⋅⋅⋅, *X*_*N* − 1_ and *X_N_* are the results of the discrete FT of the *N* input amplitudes.

The quantum circuit model of the QFT utilizes the 1‐qubit Hadamard gate

(3) $${\hat{U}}_{H}=\frac{1}{\sqrt{2}}\left[\begin{array}{cc} 1 & 1\\ 1 & -1 \end{array}\right]$$

and the 2‐qubit controlled phase gates

(4) $${\hat{U}}_{CR_{m}}=\left[\begin{array}{cccc} 1 & & & \\ & 1 & & \\ & & 1 & \\ & & & e^{2\pi i/2^{m}} \end{array}\right],$$

where *m* is a variable parameter. To simulate the QFT using an electric circuit, two essential actions must be performed. One is defining the analog of a qubit with the state of the electric circuits, and the other is certifying the electric operations on those states that can realize the function of the above quantum gates. To simulate the qubit, we define “cebit” in our circuit design. Each cebit contains 4 voltage signals, and we can get 2^*n*^ classical states to correspond to quantum states using the 4*n*voltage signals of the *n* cebits. To simulate the quantum gates, we realize the corresponding circuit gates.

**Figure 1:**
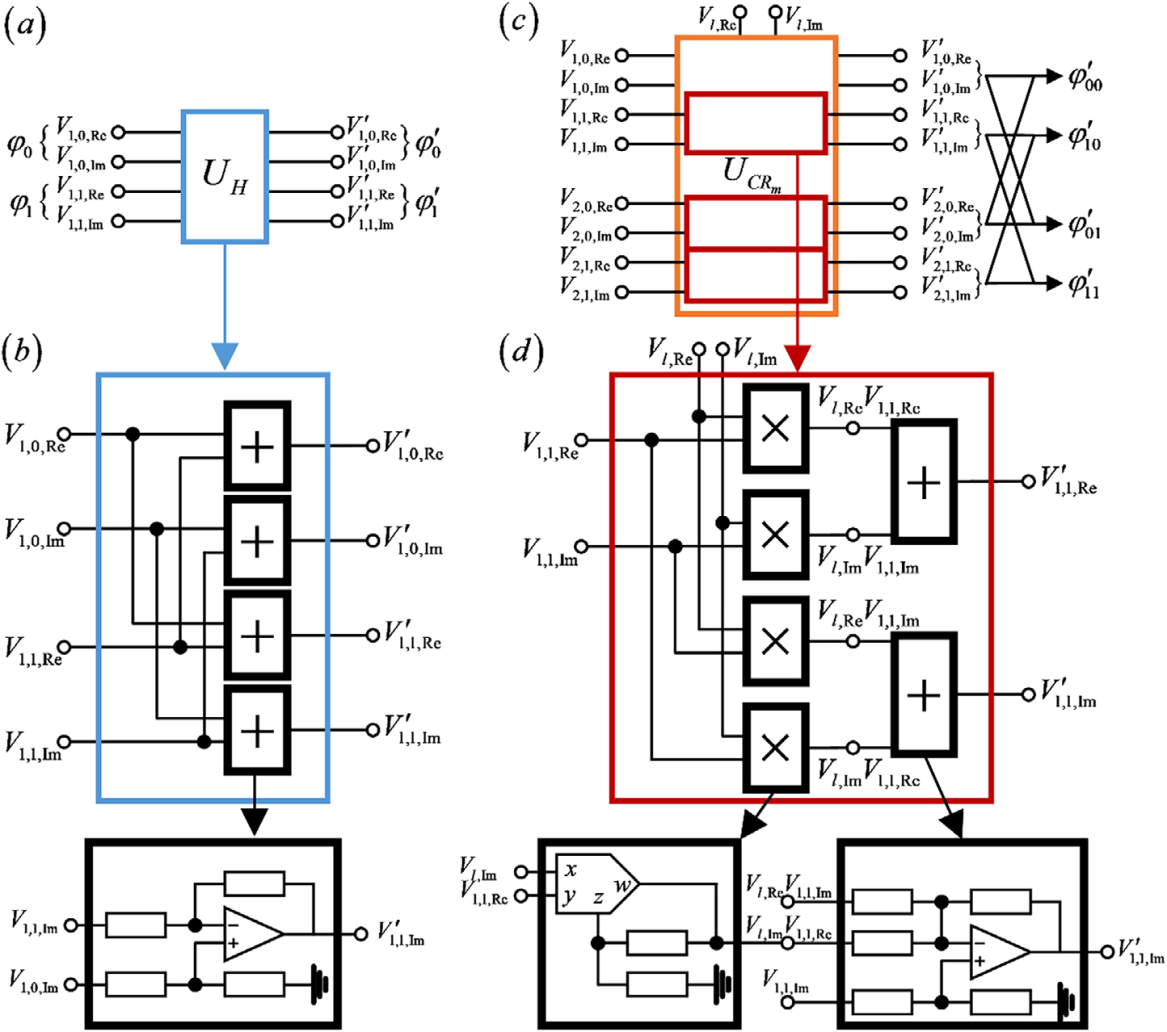
a) The diagram of the 1‐cebit gate *U_H_*. In 1‐cebit system, the amplitude φ_0_ depends on voltage signals $V_{1,0,Re}$ and $V_{1,0,Im}$, while the amplitude φ_1_ depends on voltage signals $V_{1,1,Re}$ and $V_{1,1,Im}$. b) The circuit design of the 1‐cebit gate *U_H_*. It consists of 4 adder modules. These modules share a similar design, but their specific configurations differ. One typical module includes 1 operational amplifier (represented by triangle) and 4 resistors (represented by a rectangle). c) The diagram of the 2‐cebit gate $U_{CR_{m}}$. In 2‐cebit system, the amplitude φ_00_ depends on the voltage signals $V_{1,0,Re},V_{1,0,Im},V_{2,0,Re}$ and $V_{2,0,Im}$, and so do the other amplitudes. d) The circuit design of the CR module. It consists of 4 multiplier modules (marked by “ × ”) and 2 adder modules (marked by “+ ”). Different multiplier modules share identical designs, containing 1 analog multiplier (represented by pentagon) and 2 resistor (represented by a rectangle), while adder modules differ in their circuit designs, containing 1 analog adder (represented by triangle) and varying numbers resistor (represented by a rectangle).

We first take the correspondence to the 1‐qubit gate ${\hat{U}}_{H}$ as an example. The ${\hat{U}}_{H}$ gate acts on the quantum state $\left|\phi \right\rangle =\phi_{0}\left|0\right\rangle +\phi_{1}\left|1\right\rangle =\left[\begin{array}{c} \phi_{0}\\ \phi_{1} \end{array}\right]$ as

(5) $$\left[\begin{array}{c} {\phi^{\prime }}_{0}\\ {\phi^{\prime }}_{1} \end{array}\right]={\hat{U}}_{H}\left[\begin{array}{c} \phi_{0}\\ \phi_{1} \end{array}\right]=\frac{1}{\sqrt{2}}\left[\begin{array}{c} \phi_{0}+\phi_{1}\\ \phi_{0}-\phi_{1} \end{array}\right],$$

where ϕ_0_ and ϕ_1_ represent the amplitude of the quantum state |ϕ〉, and they are transformed into ϕ′_0_ and ϕ′_1_. To implement the corresponding 1‐qubit operation in the classical circuit, we construct the circuit network *U_H_* as shown in **Figure** **1**a. The 4 input voltage signals denoted by $\vec{V_{1}}$ $=(V_{1,0,Re},$ $V_{1,0,Im},$ $V_{1,1,Re},$ $V_{1,1,Im}$)^T^, where “T” represents the matrix transpose, are used as the analog of a qubit as a whole and known as a cebit. Each component of $\vec{V_{1}}$ is a time‐dependent voltage function. To implement the function of the Hadamard gate correspondingly, the circuit network *U_H_* is required to transform the input voltage signals into the output voltage signals ($V^{\prime }={\left({V^{\prime }}_{1,0,Re},{V^{\prime }}_{1,0,Im},{V^{\prime }}_{1,1,Re},{V^{\prime }}_{1,1,Im}\right)}^{T}$, as shown by

(6) $$\left[\begin{array}{c} {V^{\prime }}_{1,0,Re}\\ {V^{\prime }}_{1,0,Im}\\ {V^{\prime }}_{1,1,Re}\\ {V^{\prime }}_{1,1,Im} \end{array}\right]=\frac{1}{\sqrt{2}}\left[\begin{array}{c} V_{1,0,Re}+V_{1,1,Re}\\ V_{1,0,Im}+V_{1,1,Im}\\ V_{1,0,Re}-V_{1,1,Re}\\ V_{1,0,Im}-V_{1,1,Im} \end{array}\right].$$

Subsequently we demonstrate that the transformation of the voltage signals can be described by the classical state |φ) corresponding to the quantum state |ϕ〉. To illustrate the relation between the classical states and the quantum states clearer, we employ the notation “|)” to denote the classical states given by cebit. In particular, the classical state of the voltage signals is expressed by

(7) $$\left.\left|\varphi \right.\right)=\varphi_{0}\left.\left|0\right.\right)+\varphi_{1}\left.\left|1\right.\right)=\left[\begin{array}{c} \varphi_{0}\\ \varphi_{1} \end{array}\right],$$

where

(8) $$\varphi_{0}=\frac{1}{T}\int_{0}^{T}dt\sum_{w=1}^{W}e^{-iw\omega t}\left(V_{1,0,Re}+iV_{1,0,Im}\right),$$

(9) $$\varphi_{1}=\frac{1}{T}\int_{0}^{T}dt\sum_{w=1}^{W}e^{-iw\omega t}\left(V_{1,1,Re}+iV_{1,1,Im}\right).$$

φ_0_ and φ_1_ represents the complex amplitudes of the classical state |φ) corresponding to the complex amplitudes of the quantum state |ϕ〉. As complex values, they are obtained from the complex voltage signal function $V_{1,0,Re}+iV_{1,0,Im}$ and $V_{1,1,Re}+iV_{1,1,Im}$ respectively. *T* = 2π/ω is the period of the voltage signals, where the frequency ω depends on the experimental setting. And the summation upper limit *W* for the index *w* is determined by the input voltage $V_{1,0,Re},V_{1,0,Im},V_{1,1,Re}$ and $V_{1,1,Im}$. We denote the highest frequency component in the spectrum of *V* as *W*ω. Since ω is known, *W* can be derived accordingly. Under the definition above, the gate *U_H_* transforms the input classical state |φ) into the output state |φ′) = φ′_0_|0) + φ′_1_|1), where

(10) $$\begin{array}{ccc} {\varphi^{\prime }}_{0} & = & \frac{1}{T}\int_{0}^{T}dt\sum_{j=1}^{W}e^{-ij\omega t}\left({V^{\prime }}_{1,0,Re}+i{V^{\prime }}_{1,0,Im}\right)\\ & = & \frac{1}{T}\int_{0}^{T}dt\sum_{j=1}^{W}e^{-ij\omega t}\frac{V_{1,0,Re}+V_{1,1,Re}+iV_{1,0,Im}+iV_{1,1,Im}}{\sqrt{2}}\\ & = & \frac{1}{T}\int_{0}^{T}dt\sum_{j=1}^{W}e^{-ij\omega t}\frac{V_{1,0,Re}+iV_{1,0,Im}}{\sqrt{2}}\\ & & +\;\frac{1}{T}\int_{0}^{T}dt\sum_{j=1}^{W}e^{-ij\omega t}\frac{V_{1,1,Re}+iV_{1,1,Im}}{\sqrt{2}}\\ & = & \frac{\varphi_{0}+\varphi_{1}}{\sqrt{2}}, \end{array}$$

and

(11) $$\varphi_{1}^{\prime }=\frac{\varphi_{0}-\varphi_{1}}{\sqrt{2}}.$$

So we have

(12) $$\left.\left|\varphi^{\prime }\right.\right)=\varphi_{0}^{\prime }\left.\left|0\right.\right)+\varphi_{1}^{\prime }\left.\left|1\right.\right)=\left[\begin{array}{c} {\varphi^{\prime }}_{0}\\ {\varphi^{\prime }}_{1} \end{array}\right]=\frac{1}{\sqrt{2}}\left[\begin{array}{c} \varphi_{0}+\varphi_{1}\\ \varphi_{0}-\varphi_{1} \end{array}\right],$$

which corresponds to the result shown in Equation (5).

Next, we briefly describe the design of the circuit network *U_H_* used to implement the transformation shown in Equation (6). As is shown in Figure 1b, *U_H_* consists of 4 analog adder/subtractor modules, which are referred to as “adder/subtractor circuit”. Since subtraction is mathematically equivalent to adding a negative value, we simply call it “adder module”, and mark the module by the black boxes with plus sign “+ ”. Each module has 2 input and 1 output, performing weighted addition operations on the input voltage signals and outputs the result. These adder modules share a similar design, but their specific configurations are different. Here, we illustrate the structure of one certain modules. This depicted module contains 1 operational amplifier (represented by triangle) and 4 resistors (represented by rectangle). The design details of the module and its calculation results are provided in the subhead “The detailed circuit design of 1‐cebit gate (adder module)” of Experimental Section.

Now we consider the correspondence 2‐qubit gate ${\hat{U}}_{CR_{m}}$. The ${\hat{U}}_{CR_{m}}$ acts on the quantum state |ϕ〉 = ϕ_00_|00〉 + ϕ_01_|01〉 + ϕ_10_|10〉 + ϕ_11_|11〉 as

(13) $$\left[\begin{array}{c} {\phi^{\prime }}_{00}\\ {\phi^{\prime }}_{01}\\ {\phi^{\prime }}_{10}\\ {\phi^{\prime }}_{11} \end{array}\right]={\hat{U}}_{CR_{m}}\left[\begin{array}{c} \phi_{00}\\ \phi_{01}\\ \phi_{10}\\ \phi_{11} \end{array}\right]=\left[\begin{array}{c} \phi_{00}\\ \phi_{01}\\ \phi_{10}\\ e^{2\pi i/2^{m}}\phi_{11} \end{array}\right],$$

where ϕ_00_,ϕ_01_,ϕ_10_ and ϕ_11_ represent the amplitude of the quantum state. To implement the corresponding 2‐qubit operation using the classical circuit, we construct the circuit network $U_{CR_{m}}$ as shown in Figure 1c. The 8 voltage signals for two cebits are denoted as $\vec{V_{k}}={\left(V_{k,0,Re},V_{k,0,Im},V_{k,1,Re},V_{k,1,Im}\right)}^{T}$(*k* = 1, 2). In addition, this classical gate contains 2 fixed inputs $V_{l,Re}$ and $V_{l,Im}$, which multiply with these component voltage signals. The circuit network $U_{CR_{m}}$ transforms these voltage signals into $\vec{{V^{\prime }}_{k}}=(V_{k,0,Re}^{\prime },V_{k,0,Im}^{\prime },V_{k,1,Re}^{\prime },$ $V_{k,1,Im}^{\prime }$)^T^, and we have

(14) $$\begin{array}{c} \left\{\begin{array}{c} \left[\begin{array}{c} {V^{\prime }}_{1,0,Re}\\ {V^{\prime }}_{1,0,Im}\\ {V^{\prime }}_{1,1,Re}\\ {V^{\prime }}_{1,1,Im} \end{array}\right]=\left[\begin{array}{c} V_{1,0,Re}\\ V_{1,0,Im}\\ V_{l,Re}V_{1,1,Re}-V_{l,Im}V_{1,1,Im}\\ V_{l,Re}V_{1,1,Im}+V_{l,Im}V_{1,1,Re} \end{array}\right],\\ \left[\begin{array}{c} {V^{\prime }}_{2,0,Re}\\ {V^{\prime }}_{2,0,Im}\\ {V^{\prime }}_{2,1,Re}\\ {V^{\prime }}_{2,1,Im} \end{array}\right]=\left[\begin{array}{c} V_{2,0,Re}+V_{l,Re}V_{2,0,Re}-V_{l,Im}V_{2,0,Im}\\ V_{2,0,Im}+V_{l,Re}V_{2,0,Im}+V_{l,Im}V_{2,0,Re}\\ V_{2,1,Re}+\cos\left(2\pi /2^{m}\right)F_{Re}-\sin\left(2\pi /2^{m}\right)F_{Im}\\ V_{2,1,Im}+\sin\left(2\pi /2^{m}\right)F_{Re}+\cos\left(2\pi /2^{m}\right)F_{Im} \end{array}\right]. \end{array}\right.\\ \left(F_{Re}=V_{l,Re}V_{2,1,Re}-V_{l,Im}V_{2,1,Im},F_{Im}=V_{l,Re}V_{2,1,Im}+V_{l,Im}V_{2,1,Re}\right) \end{array}$$

The classical state |φ) can be expressed by the voltage signals using the above definitions

(15) $$\left.\left|\varphi \right.\right)=\varphi_{00}\left.\left|00\right.\right)+\varphi_{01}\left.\left|01\right.\right)+\varphi_{10}\left.\left|10\right.\right)+\varphi_{11}\left.\left|11\right.\right)=\left[\begin{array}{c} \varphi_{00}\\ \varphi_{01}\\ \varphi_{10}\\ \varphi_{11} \end{array}\right],$$

where

(16) $$\varphi_{s_{1},s_{2}}=\frac{1}{T}\int_{0}^{T}dt\sum_{j=1}^{W}e^{-ij\omega t}F_{1,s_{1}}*F_{2,s_{2}}.$$

Here $\varphi_{s_{1},s_{2}}$ represents the amplitude of the classical state, and the subscript *s*_1_ and *s*_2_ take the value 0 or 1, the functions represent

(17) $$F_{1,s}=V_{1,s,Re}+iV_{1,s,Im},F_{2,s}=V_{2,s,Re}+iV_{2,s,Im},$$

and the symbol “*” is the convolution operation, for the periodic functions *f*_1_ and *f*_2_ with period *T*, we have

(18) $$f_{1}*f_{2}=\frac{1}{T}\int_{0}^{T}d\tau f_{1}\left(\tau \right)f_{2}\left(t-\tau \right).$$

In general convolution operations, the integration limits range from −  ∞ to + ∞, however, for periodic sine and cosine function, the integration limits are confined to the interval [0, *T*]. Both Equations (16) and (18) have *T* as the upper integration limits, which is the period of the voltage signals.

As an example, if we take *s*_1_ = *s*_2_ = 0, then

(19) $$\begin{array}{ccc} F_{1,0}*F_{2,0} & = & \frac{1}{T}\int_{0}^{T}d\tau \left[\left(V_{1,0,Re}\left(\tau \right)+iV_{1,0,Im}\left(\tau \right)\right)\right.\\ & & \left.\times \;\left(V_{2,0,Re}\left(t-\tau \right)+iV_{2,0,Im}\left(t-\tau \right)\right)\right]. \end{array}$$

So the amplitude φ_00_ depends on the complex voltage signals $V_{1,0,Re}+iV_{1,0,Im}$ and $V_{2,0,Re}+iV_{2,0,Im}$, as the other amplitudes do.

Under the representation scheme in the above, the gate $U_{CR_{m}}$ transforms the input classical state |φ) into the output state

(20) $$\left.\left|\varphi^{\prime }\right.\right)=\left[\begin{array}{c} {\varphi^{\prime }}_{00}\\ {\varphi^{\prime }}_{01}\\ {\varphi^{\prime }}_{10}\\ {\varphi^{\prime }}_{11} \end{array}\right]=\left[\begin{array}{c} \varphi_{00}\\ \varphi_{01}\\ \varphi_{10}\\ e^{2\pi i/2^{m}}\varphi_{11} \end{array}\right]$$

It corresponds to the result in Equation (13), which describes the action of the 2‐qubit quantum gate ${\hat{U}}_{CR_{m}}$ on the quantum state. The proof details are provided in the subhead “The detailed circuit design and the output calculation of CR module” of Experimental Section.

Next, we describe the design of the circuit network $U_{CR_{m}}$ used to implement the transformation in Equation (14). As shown in Figure 1c, $U_{CR_{m}}$ it consists of 3 modules (red boxes), which we refer to as “CR modules”. The first CR module receives input voltage signals $V_{1,1,Re}$ and $V_{1,1,Im}$, the second incorporates $V_{2,0,Re}$ and $V_{2,0,Im}$ among its inputs, while the third operates with $V_{2,1,Re}$ and $V_{2,1,Im}$.The remaining 2 input signals $V_{1,0,Re}$ and $V_{1,0,Im}$ remain fixed. In addition, $V_{l,Re}$ and $V_{l,Im}$ are externally applied single‐frequency cosine and sine signals, respectively. Their frequency is set to match the highest spectral component within the combined spectra of all the voltage signals.

Figure 1d illustrates a certain CR module whose outputs $V_{1,1,Re}^{\prime }$ and $V_{1,1,Im}^{\prime }$ are defined by Equation (14). To achieve $V_{1,1,Re}^{\prime }$, we multiply $V_{l,Re}$ and $V_{1,1,Re}$ through the multiplier module (marked by “ × ”) to obtain $V_{l,Re}V_{1,1,Re}$, and multiply $V_{l,Im}$ and $V_{1,1,Im}$ to obtain $V_{l,Im}V_{1,1,Im}$. These two resultant signals are then subtracted through the adder module (marked by “+ ”), producing the final output $V_{l,Re}V_{1,1,Re}-V_{l,Im}V_{1,1,Im}$. The left component voltage signals follow the analogous procedure, but the adder modules in these cases incorporate additional input signals.

One CR module consists of 4 multiplier modules and 2 adder modules, and we illustrate one certain multiplier module and one certain adder module. Generally, each multiplier module contains 1 analog multiplier (represented by pentagon) and 2 resistor (represented by rectangle), while each adder modules contains 1 analog adder (represented by triangle) and varying numbers resistor (represented by rectangle), with the resistor count dependent on the number of input signals to the analog adder. The design details of the module and its calculation results are provided in the subhead “The detailed circuit design and the output calculation of CR module” of Experimental Section.

It is well known that generating quantum entanglements is the key function of the 2‐qubit quantum gate, therefore our 2‐cebit circuit gate needs to have analogous effects. Now we analyze the classical correlation in our circuit design from two aspects. Mathematically, it manifests in the operational equivalence between the classical gates described in Equation (20) and the quantum gates in Equation (13). Physically, since entanglement typically originates from interactions that can be mathematically characterized through multiplication operation, the multiplication introduced in our circuit generates similar interaction‐like effects. In the following section, we will provide experimental evidence.

After the discussion of the circuit correspondence of the quantum gates in low‐qubit systems, we now consider scaling the system up to *n* cebit, which can be thought of as an analog of an *n*‐qubit system. When the number of cebit increases to *n*, the implementation of the two types of circuit gates is required to be generalized properly based on the above designs. Since the gates depend on the definition of the classical states obtained by the voltage signals, we first address how the definition evolves when scaling from a 2‐cebit to an *n*‐cebit system.

In the *n*‐cebit circuit, the input voltage signals for each cebit are denoted as $\vec{V_{k}}={\left(V_{k,0,Re},V_{k,0,Im},V_{k,1,Re},V_{k,1,Im}\right)}^{T}$(*k* = 1, 2, ⋅⋅⋅, *n*). The classical state is obtained from the complex voltage signals as

(21) $$\left.\left|\varphi \right.\right)=\sum_{s_{1},s_{2},\text{…},s_{n}}\varphi_{s_{1}s_{2}\text{…}s_{n}}\left.\left|s_{1}s_{2}\text{…}s_{n}\right.\right),\;\;\;\;\;\;\;$$

(22) $$\varphi_{s_{1}s_{2}\text{…}s_{n}}=\frac{1}{T}\int_{0}^{T}dt\sum_{j=1}^{W}e^{-ij\omega t}\prod_{k=1}^{n}*\left(V_{k,s_{k},Re}+iV_{k,s_{k},Im}\right),$$

where the subscripts *s_k_* take the value 0 or 1, so φ has 2^*n*^ subscripts. We denote the successive convolution operation by the symbol “∏*”, which is defined as

(23) $$\prod_{k=1}^{n}*\left(f_{k}\right)=f_{1}*f_{2}*\text{…}*f_{n}.$$

Following the discussion on the evolution of the definition of the classical states, we now address the modifications to the *U_H_* gate and the $U_{CR_{m}}$ gate when scaling to the *n*‐cebit system. The design of *U_H_* gate remains consistent in the *n*‐cebit system. To implement *U_H_* gate on the *k*_0_‐th cebit, we feed the input voltage signals of the *k*_0_‐th cebit into the *U_H_* gate, while we keep the voltage signals of all other cebits unchanged. The *U_H_* gate transforms the classical state into

(24) $$\left[\begin{array}{c} {\varphi^{\prime }}_{s_{k_{0}}=0}\\ {\varphi^{\prime }}_{s_{k_{0}}=1} \end{array}\right]=\frac{1}{\sqrt{2}}\left[\begin{array}{c} \varphi_{s_{k_{0}}=0}+\varphi_{s_{k_{0}}=1}\\ \varphi_{s_{k_{0}}=0}-\varphi_{s_{k_{0}}=1} \end{array}\right].$$

This expression corresponds to the result of extending Equation (12) to the *n*‐cebit system. Detailed calculations are provided in the subhead “The Detailed Circuit Design of 1‐Cebit Gate (Adder Module)” of Experimental Section This expression also corresponds to the action result of the 1‐qubit Hadamard gate ${\hat{U}}_{H}$ in the *n*‐qubit system

(25) $$\left[\begin{array}{c} {\phi^{\prime }}_{s_{k_{0}}=0}\\ {\phi^{\prime }}_{s_{k_{0}}=1} \end{array}\right]={\hat{U}}_{H}\left[\begin{array}{c} \phi_{s_{k_{0}}=0}\\ \phi_{s_{k_{0}}=1} \end{array}\right]=\frac{1}{\sqrt{2}}\left[\begin{array}{c} \phi_{s_{k_{0}}=0}+\phi_{s_{k_{0}}=1}\\ \phi_{s_{k_{0}}=0}-\phi_{s_{k_{0}}=1} \end{array}\right],$$

where $\varphi_{s_{k_{0}}=0}$ should be understood as selecting the components when the subscript $s_{k_{0}}=0$ from the entire set of $\varphi_{s_{1}s_{2}\text{…}s_{n}}$, and then forming a column vector composed of these selected components. And so are $\varphi_{s_{k_{0}}=1}$, $\phi_{s_{k_{0}}=0}$ and $\phi_{s_{k_{0}}=1}$.

**Figure 2:**
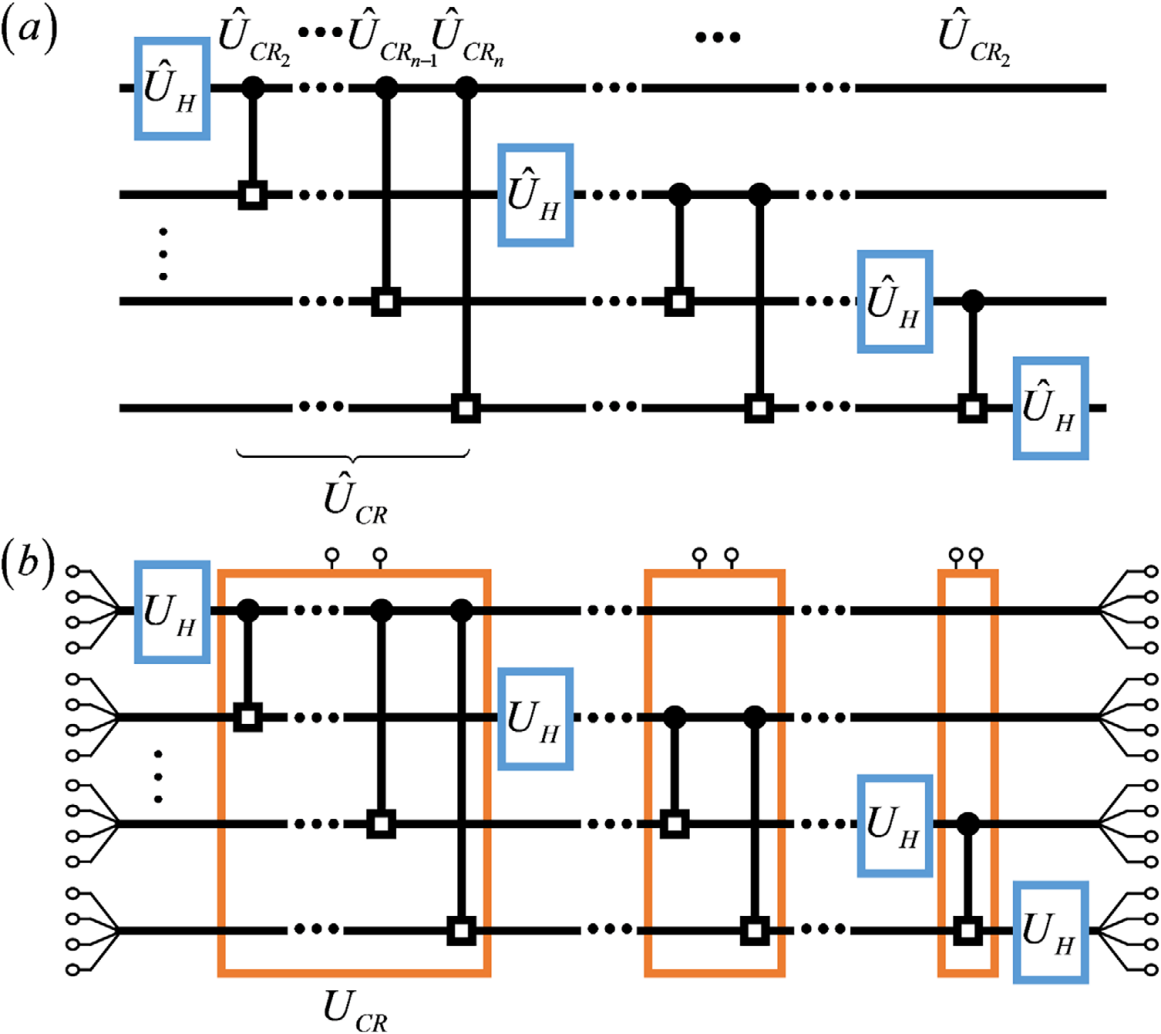
a) Quantum circuit for the *n*‐qubit QFT. b) Classical circuit simulating the *n*‐qubit QFT.

During the extension of the $U_{CR_{m}}$ gate to the *n*‐cebit system, we uncover an intriguing property, that the circuit system can efficiently implement the classical correlations among multiple cebits. We first talk about the multi‐qubit entanglement in quantum system. As is shown in **Figure** **2**a, the multi‐qubit entangled operation ${\hat{U}}_{CR}$ in QFT is constructed by cascading multiple ${\hat{U}}_{CR_{m}}$ gate

(26) $${\hat{U}}_{CR}={\hat{U}}_{k_{1}\to k_{1}+1}{\hat{U}}_{k_{1}\to k_{1}+2}\text{…}{\hat{U}}_{k_{1}\to n}.$$

Here ${\hat{U}}_{k\to k+l}$(*l* = 1, 2, ⋅⋅⋅, *n* − *k*) represents a controlled‐phase ${\hat{U}}_{CR_{m}}$ gate with the *k*‐th qubit being the control qubit and the (*k* + *l*)‐th qubit being the target qubit, and the parameter *m* equals to *l* + 1. The qubits in ${\hat{U}}_{CR_{m}}$ gate are divided into three part *k*_0_,*k*_1_ and *k*_2_ as followed. In this multi‐qubit entanglement scheme, these ${\hat{U}}_{CR_{m}}$ gates share the same control qubit called *k*_1_. And the (*k*_1_ + 1)‐thto the *n*‐th qubit are collectively denoted as *k*_2_, since these qubit are the target qubit for one controlled‐phase gate in ${\hat{U}}_{CR}$. The remain qubits are labeled as *k*_0_, spanning from 1 to *k*_1_ − 1. In quantum system, these gates rely on distinct entanglement correlations between different qubits, and is hard to be implemented with a single quantum operation. However, in classical circuit, such classical correlations among multi‐cebit can be realized straightforwardly. This is achieved using a single *U_CR_* gate as is shown in Figure 2b, and it transforms these voltage signals into

(27) $$\begin{array}{c} \begin{array}{c} \left\{\begin{array}{c} \left[\begin{array}{c} {V^{\prime }}_{k_{0},0,Re}\\ {V^{\prime }}_{k_{0},0,Im}\\ {V^{\prime }}_{k_{0},1,Re}\\ {V^{\prime }}_{k_{0},1,Im} \end{array}\right]=\left[\begin{array}{c} V_{k_{0},0,Re}+V_{l,Re}V_{k_{0},0,Re}-V_{l,Im}V_{k_{0},0,Im}\\ V_{k_{0},0,Im}+V_{l,Re}V_{k_{0},0,Im}+V_{l,Im}V_{k_{0},0,Re}\\ V_{k_{0},1,Re}+V_{l,Re}V_{k_{0},1,Re}-V_{l,Im}V_{k_{0},1,Im}\\ V_{k_{0},1,Im}+V_{l,Re}V_{k_{0},1,Im}+V_{l,Im}V_{k_{0},1,Re} \end{array}\right],\\ \left[\begin{array}{c} {V^{\prime }}_{k_{1},0,Re}\\ {V^{\prime }}_{k_{1},0,Im}\\ {V^{\prime }}_{k_{1},1,Re}\\ {V^{\prime }}_{k_{1},1,Im} \end{array}\right]=\left[\begin{array}{c} V_{k_{1},0,Re}\\ V_{k_{1},0,Im}\\ V_{l,Re}V_{k_{1},1,Re}-V_{l,Im}V_{k_{1},1,Im}\\ V_{l,Re}V_{k_{1},1,Im}+V_{l,Im}V_{k_{1},1,Re} \end{array}\right],\\ \left[\begin{array}{c} {V^{\prime }}_{k_{2},0,Re}\\ {V^{\prime }}_{k_{2},0,Im}\\ {V^{\prime }}_{k_{2},1,Re}\\ {V^{\prime }}_{k_{2},1,Im} \end{array}\right]=\left[\begin{array}{c} V_{k_{2},0,Re}+V_{l,Re}V_{k_{2},0,Re}-V_{l,Im}V_{k_{2},0,Im}\\ V_{k_{2},0,Im}+V_{l,Re}V_{k_{2},0,Im}+V_{l,Im}V_{k_{2},0,Re}\\ V_{k_{2},1,Re}+\cos\left(2\pi /2^{m}\right)F_{Re}-\sin\left(2\pi /2^{m}\right)F_{Im}\\ V_{k_{2},1,Im}+\sin\left(2\pi /2^{m}\right)F_{Re}+\cos\left(2\pi /2^{m}\right)F_{Im} \end{array}\right], \end{array}\right.\\ \left(F_{Re}=V_{l,Re}V_{k_{2},1,Re}-V_{l,Im}V_{k_{2},1,Im},F_{Im}=V_{l,Re}V_{k_{2},1,Im}+V_{l,Im}V_{k_{2},1,Re}\right) \end{array} \end{array}$$

Similar to the qubits in Equation (26), the cebits are also divided into three parts labeled as *k*_0_,*k*_1_ and *k*_2_, where *k*_0_ spans from 1 to *k*_1_ − 1 and *k*_2_ ranges from *k*_1_ + 1 to *n*. Here $V_{l,Re}$ and $V_{l,Im}$ are externally applied single‐frequency cosine and sine signals, respectively. Their frequency is set to match the highest spectral component within the combined spectra of all the voltage signals. By comparing Equation (27) with Equation (14), it can be observed that the fundamental construction schemes of the *U_CR_* gate and the $U_{CR_{m}}$ gate are similar, and both are composed of CR modules. In the *U_CR_* gate, each *k*_0_‐th cebit contains 2 CR modules, each *k*_1_‐th contains 1 CR module, and each *k*_2_‐th cebit contains 2 CR modules. Using the definition of the classical states in Equation (22), the result of the *U_CR_* gate corresponds to the ${\hat{U}}_{CR}$ gate. The detailed computing process is provided in Method (B).

Using the circuit gates mentioned above, the circuit simulation of the QFT can be realized. As is shown in Figure 2a, the *n*‐qubit QFT can be implemented using the quantum ${\hat{U}}_{H}$ and ${\hat{U}}_{CR_{m}}$ gates, and the omitted quantum swap gate. By replacing the quantum gates in the quantum circuit with our corresponding circuit designs, the classical circuit simulation is achieved, as is shown in Figure 2b. For visual simplicity, the 4 input signals for each cebit are merged into a single line. In circuit simulation, we omit the swap gates too.

Now, we briefly describe the QFT simulation in the classical circuit using the classical basic state as an example. Since the definition of the classical states obtained by the voltage signals has been established, we no longer describe the voltage signals, but instead focus solely on the evolution of the classical state. The input classical basic state is expressed as

(28) $$\begin{array}{c} \left.\left|K\right.\right)=\left.\left|\bar{K_{1}K_{2}\text{…}K_{n}}\right.\right)\\ \;\;\;\;=\left.\left|K_{1}\right.\right)⊗\left.\left|K_{2}\right.\right)⊗\text{…}⊗\left.\left|K_{n}\right.\right), \end{array}$$

where the decimal representation *K* is rewritten as the binary representation $\bar{K_{1}K_{2}\text{…}K_{n}}$.

At first, the *U_H_* gate acts on the input state as

(29) $$\left\{\begin{array}{cc} \frac{\left.\left|0\right.\right)+\left.\left|1\right.\right)}{\sqrt{2}}⊗\left.\left|\bar{K_{2}\text{…}K_{n}}\right.\right), & K_{1}=0,\\ \frac{\left.\left|0\right.\right)-\left.\left|1\right.\right)}{\sqrt{2}}⊗\left.\left|\bar{K_{2}\text{…}K_{n}}\right.\right), & K_{1}=1. \end{array}\right.$$

We rewrite it as

(30) $$\frac{\left.\left|0\right.\right)+e^{i2\pi \bar{0.K_{1}}}\left.\left|1\right.\right)}{\sqrt{2}}⊗\left.\left|\bar{K_{2}\text{…}K_{n}}\right.\right).$$

Here we employ the binary fractional notation

(31) $$\bar{0.a_{1}a_{2}\text{…}a_{n}}=\sum_{j=1}^{n}2^{-j}a_{j}.$$

For example, $\bar{0.K_{1}}=K_{1}/2$.

Next, the *U_CR_* gate acts on the classical state as

(32) $$\frac{\left.\left|0\right.\right)+e^{i2\pi \bar{0.K_{1}K_{2}\text{…}K_{n}}}\left.\left|1\right.\right)}{\sqrt{2}}⊗\left.\left|\bar{K_{2}\text{…}K_{n}}\right.\right)=\left.\left|{K^{\prime }}_{1}\right.\right)⊗\left.\left|\bar{K_{2}\text{…}K_{n}}\right.\right)$$

The component |1) of the classical state |*K*_1_) has a $2\pi /2^{K_{k}}$ phase shift controlled by the *k*‐th cebit, transforming |*K*_1_) into |*K*′_1_). Subsequent gates similarly transform |*K*_2_) into |*K*′_2_), transform |*K*_3_) into |*K*′_3_), and so on, but these gates no longer change the state |*K*′_1_). And we have

(33) $$\left.\left|{K^{\prime }}_{k}\right.\right)=\frac{\left.\left|0\right.\right)+e^{i2\pi \bar{0.K_{k}K_{k+1}\text{…}K_{n}}}\left.\left|1\right.\right)}{\sqrt{2}}.$$

After the SWAP gate, the final circuit output is

(34) $$\mathrm{SWAP}\left(\left.\left|\bar{{K^{\prime }}_{1}{K^{\prime }}_{2}\text{…}{K^{\prime }}_{n}}\right.\right)\right)=\left.\left|\bar{{K^{\prime }}_{n}{K^{\prime }}_{n-1}\text{…}{K^{\prime }}_{1}}\right.\right).$$

Now we consider the amplitude of one basic state $|\bar{J_{1}J_{2}\text{…}J_{n}})$. Each *J_j_* term induces a phase shift Δθ_*j*_ = $J_{j}2\pi \bar{0.K_{n-j+1}\text{…}K_{n}}$, so the phase shift of $|\bar{J_{1}J_{2}\text{…}J_{n}})$ is

(35) $$\begin{array}{c} \sum_{j=1}^{n}\Delta \theta_{j}=2\pi \sum_{j=1}^{n}J_{j}\bar{0.K_{n-j+1}\text{…}K_{n}}\\ \;\;\;\equiv 2\pi \sum_{j=1}^{n}J_{j}\bar{0.K_{n-j+1}\text{…}K_{n}}+2A\pi \\ \;\;\;=2\pi \sum_{j=1}^{n}J_{j}\bar{K_{1}K_{2}\text{…}K_{n-j}.K_{n-j+1}\text{…}K_{n}}\\ \;\;\;=2\pi 2^{n}\sum_{j=1}^{n}J_{j}2^{-j}\sum_{k=1}^{n}2^{n-j}2^{-k}K_{k}\\ \;\;\;=2\pi 2^{n}\bar{0.J_{1}\text{…}J_{n}}\bar{0.K_{1}\text{…}K_{n}}\\ \;\;\;=2\pi KJ/N. \end{array}$$

Here

(36) $$A=\sum_{j=1}^{n}J_{j}\bar{K_{1}K_{2}\text{…}K_{n-j}}$$

is an integer. The symbol “ ≡ ” in Equation (35) means that the two sides of the symbol differ by an integer multiple of 2π, thus they induce identical phase shifts. Also the phase shift in Equation (35) is equivalent to the phase shift in Equation (1). Based on the linear properties of the circuit system, the correspondence to Equation (2) arises naturally, and we successfully realize the QFT simulation in the classical circuit.

We emphasize that the speed‐up in QFT arises from quantum entanglement, which enables a single quantum gate to process all information contained in a quantum state in parallel, thereby drastically reducing computing resource demands. Analogously, our circuit design achieves a similar parallel speed‐up by introducing a novel definition of the classical states based on correlated voltage signals, emulating classical correlations. This allows a single analog circuit component to efficiently process all information in parallel. For example, in the 2‐cebit circuit simulation of QFT, the input classical state

(37) $$\left.\left|0\right.\right)⊗\frac{\left.\left|0\right.\right)+\left.\left|1\right.\right)}{\sqrt{2}}$$

is transformed into the output state

(38) $$\frac{\left.2\left|00\right.\right)+\left.\left(1-i\right)\left|01\right.\right)+\left.\left(1+i\right)\left|11\right.\right)}{2\sqrt{2}}$$

which can be further rotated into a classical state $\frac{|00)+|11)}{\sqrt{2}}$ corresponding to a quantum Bell state $\frac{\left|00\rangle +|11\right\rangle }{\sqrt{2}}$ via two 1‐cebit gates acts on the 1‐st and the 2‐nd cebit respectively

(39) $$\frac{1}{\sqrt{2}}\left[\begin{array}{cc} 1 & -1\\ 1 & 1 \end{array}\right]⊗\frac{1}{\sqrt{2}}\left[\begin{array}{cc} 1-i & -1+i\\ 1+i & 1-i \end{array}\right].$$

Therefore, it is able to exhibit similar properties analogous to the quantum Bell state. The experimental demonstrations are given in the following section. This result shows the presence of the entanglement‐like correlation in the circuit system, and reveals the intrinsic source of the speed‐up effect in our circuit simulation scheme.

At the end of this section, we discuss the computational complexity of our circuit design. In quantum system, computational complexity is considered proportional to the number of quantum gates. Similarly in classical system, computational complexity should depend on the number of basic computing components in the circuit. However, we emphasize that multiple circuit components can be integrated into a single component, and such an integrated component clearly cannot be regarded as a basic computing component. Therefore, to start it, we need to define the basic computing component in our circuit design.

The definition of the basic computing component is based on the mathematical operations it performs. In our circuit design, we only use 3 kinds of basic computing components. The first is the wire, performing copy operation that maps one function *f* into two identical functions *f*. The second is the analog adder, performing addition (subtraction) operation that maps two functions *f*_1_ and *f*_2_ into one function *f*_1_ ± *f*_2_. The last is the analog multiplier, performing a multiplication operation that maps two functions *f*_1_ and *f*_2_ into one function *f*_1_ × *f*_2_. Thus, the computational complexity in our circuit design is determined by the number of the basic computing components employed.

Our *n*‐cebit circuit design consists of *n U_H_* gate and *n* − 1 *U_CR_* gate. Each *U_H_* gate contains 4 analog adders, while each *U_CR_* gate contains *O*(*n*) analog multipliers and *O*(*n*) analog adders. Consequently, an *n*‐cebit simulation circuit requires a total of *O*(*n* ^2^) fundamental analog circuit components. This matches the *O*(*n* ^2^) quantum gates needed for the *n*‐qubit QFT quantum circuit, and outperforms the *O*(*n*2^*n*^) = *O*(*N*log *N*) elementary operations of FFT.

After comparing the algorithm complexity among the circuit scheme, QFT and FFT, we additionally consider the processing time required per individual circuit component to compare these FT scheme in terms of process time. In our design, a circuit component operates on the voltage signals composed of sine and cosine voltage signals of different frequencies. The period of the voltage signals is denoted as *T* = 2π/ω as is in the above text, where the frequency ω is a fixed parameter set before the circuit is built. Although the output signal of the circuit component contains more frequency signals, its period remains *T*. According to Equation (22), we only need to extract a signal segment of duration *T* from each voltage signal to perform computation. The voltage signal outside the interval from time 0 to time *T* is redundant and should not be counted in the processing time. Consequently, the total complexity of this circuit design can be calculated as the processing time *T* required by the circuit components multiplied by the algorithm complexity *O*(*n* ^2^), and its order remains at the order of *O*(*n* ^2^). That is, the computation time of this circuit design scales proportionally with the hardware complexity of the circuit.

In next section, we present several circuit simulation experiments of simulating QFT to validate the correctness of our circuit design. We also experimentally verify the existence of the correspondence between the classical correlation within the designed circuit and the quantum entanglement in the quantum system.

## 3 Experimental Demonstration of the Circuit Simulation for QFT

**Figure 3:**
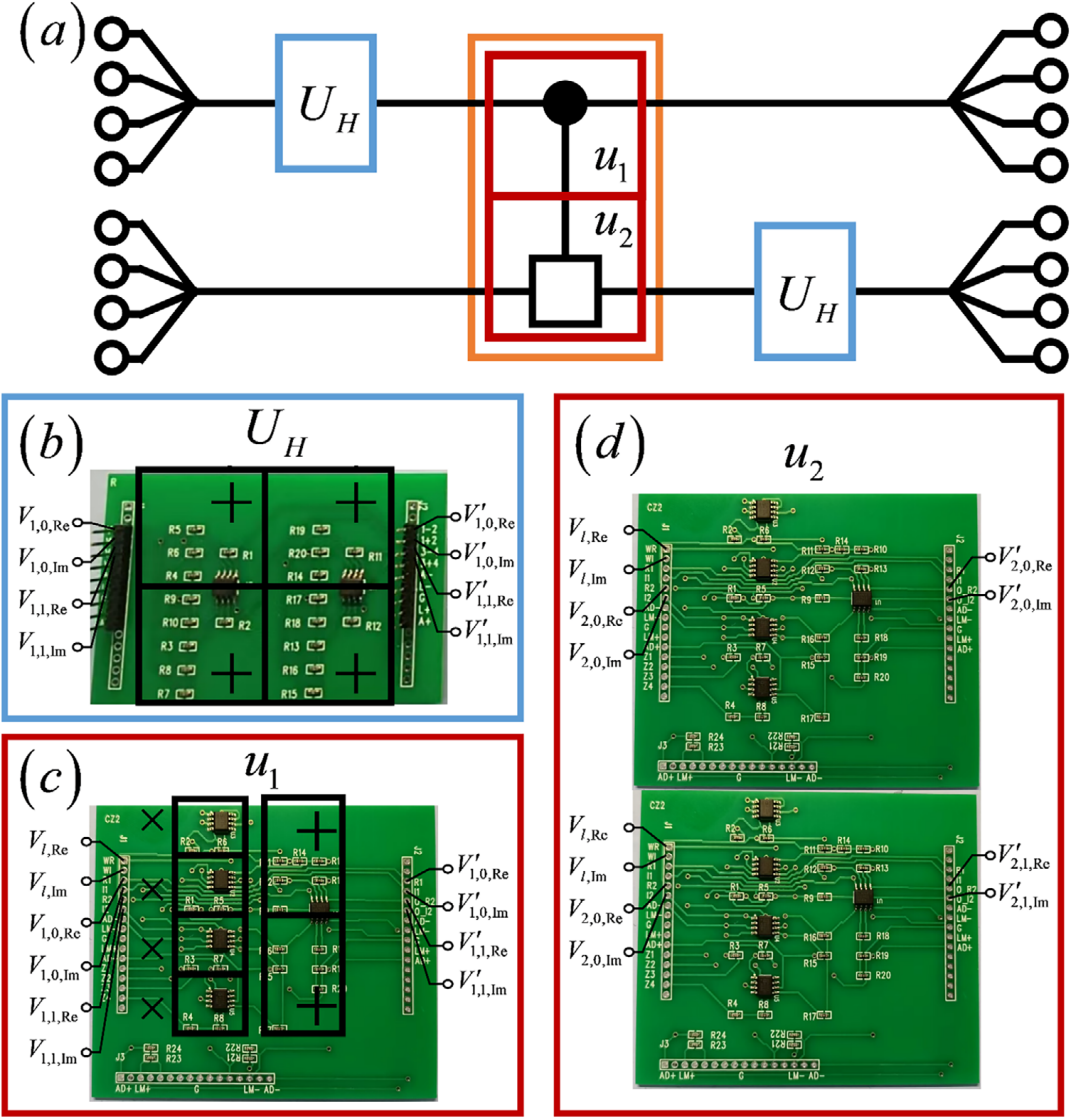
a) The schematic diagram of the 2‐cebit simulation circuit. b) The PCB layouts for the *U_H_* gate. c,d) The PCB layouts for the $U_{CR_{2}}$ gate.

We begin with the circuit simulation of the 2‐qubit QFT. The schematic diagram of the 2‐cebit simulation circuit is shown in **Figure** **3**a, consisting of 2 *U_H_* gates and 1 $U_{CR_{2}}$ gate. The $U_{CR_{2}}$ gate includes 2 submodules *u*_1_ and *u*_2_ (marked by the red boxes). The *u*_1_submodule acts on the control cebit and contains 1 CR module, while the *u*_2_submodule acts on the target cebit and contains 2 CR modules. The corresponding PCB layouts for these circuit gates are provided in Figure 3b–d. On the PCB boards, we mark the adder modules by “+ ” and the multiplier modules by “ × ”, consistent with the designs in Figure 1b,d. In these modules, the operational amplifiers used are model LM358, with each LM358 chip integrating 2 operational amplifiers. The analog multipliers employed are model AD633. The initial voltage signals in the experiment are generated using an FY2300 signal generator.

We validate our circuit design using the following 5 input states in the 2‐cebit system. These input states correspond to the initial voltage signal configurations listed below (voltage unit is V, and ω is set to 1kHz).

(40) $$\left.\left|00\right.\right):\left\{\begin{array}{c} V_{1,0,Re}=V_{2,0,Re}=\cos\left(\omega t\right),\\ V_{1,0,Im}=V_{2,0,Im}=\sin\left(\omega t\right),\\ V_{1,1,Re}=V_{1,1,Im}=V_{2,1,Re}=V_{2,1,Im}=0, \end{array}\right.$$

(41) $$\left.\left|01\right.\right):\left\{\begin{array}{c} V_{1,0,Re}=V_{2,1,Re}=\cos\left(\omega t\right),\\ V_{1,0,Im}=V_{2,1,Im}=\sin\left(\omega t\right),\\ V_{1,1,Re}=V_{1,1,Im}=V_{2,0,Re}=V_{2,0,Im}=0, \end{array}\right.$$

(42) $$\left.\left|10\right.\right):\left\{\begin{array}{c} V_{1,0,Re}=V_{1,0,Im}=V_{2,1,Re}=V_{2,1,Im}=0,\\ V_{1,1,Re}=V_{2,0,Re}=\cos\left(\omega t\right),\\ V_{1,1,Im}=V_{2,0,Im}=\sin\left(\omega t\right), \end{array}\right.$$

(43) $$\;\left.\left|11\right.\right):\left\{\begin{array}{c} V_{1,0,Re}=V_{1,0,Im}=V_{2,0,Re}=V_{2,0,Im}=0,\\ V_{1,1,Re}=V_{2,1,Re}=\cos\left(\omega t\right),\\ V_{1,1,Im}=V_{2,1,Im}=\sin\left(\omega t\right), \end{array}\right.$$

(44) $$\left.\left|0+\right.\right):\left\{\begin{array}{c} V_{1,0,Re}=\cos\left(\omega t\right),\\ V_{1,0,Im}=\sin\left(\omega t\right),\\ V_{1,1,Re}=V_{1,1,Im}=0,\\ V_{2,0,Re}=V_{2,1,Re}=\cos\left(\omega t\right)/\sqrt{2},\\ V_{2,0,Im}=V_{2,1,Im}=\sin\left(\omega t\right)/\sqrt{2}, \end{array}\right.\;\;\;\;$$

where we define

(45) $$\begin{array}{ccc} \left.\left|+\right.\right) & = & \frac{\left.\left|0\right.\right)+\left.\left|1\right.\right)}{\sqrt{2}},\left.\left|-\right.\right)=\frac{\left.\left|0\right.\right)-\left.\left|1\right.\right)}{\sqrt{2}},\left.\left|L\right.\right)=\frac{\left.\left|0\right.\right)+i\left.\left|1\right.\right)}{\sqrt{2}},\left.\left|R\right.\right)\\ & & =\frac{\left.\left|0\right.\right)-i\left.\left|1\right.\right)}{\sqrt{2}}. \end{array}$$

**Figure 4:**
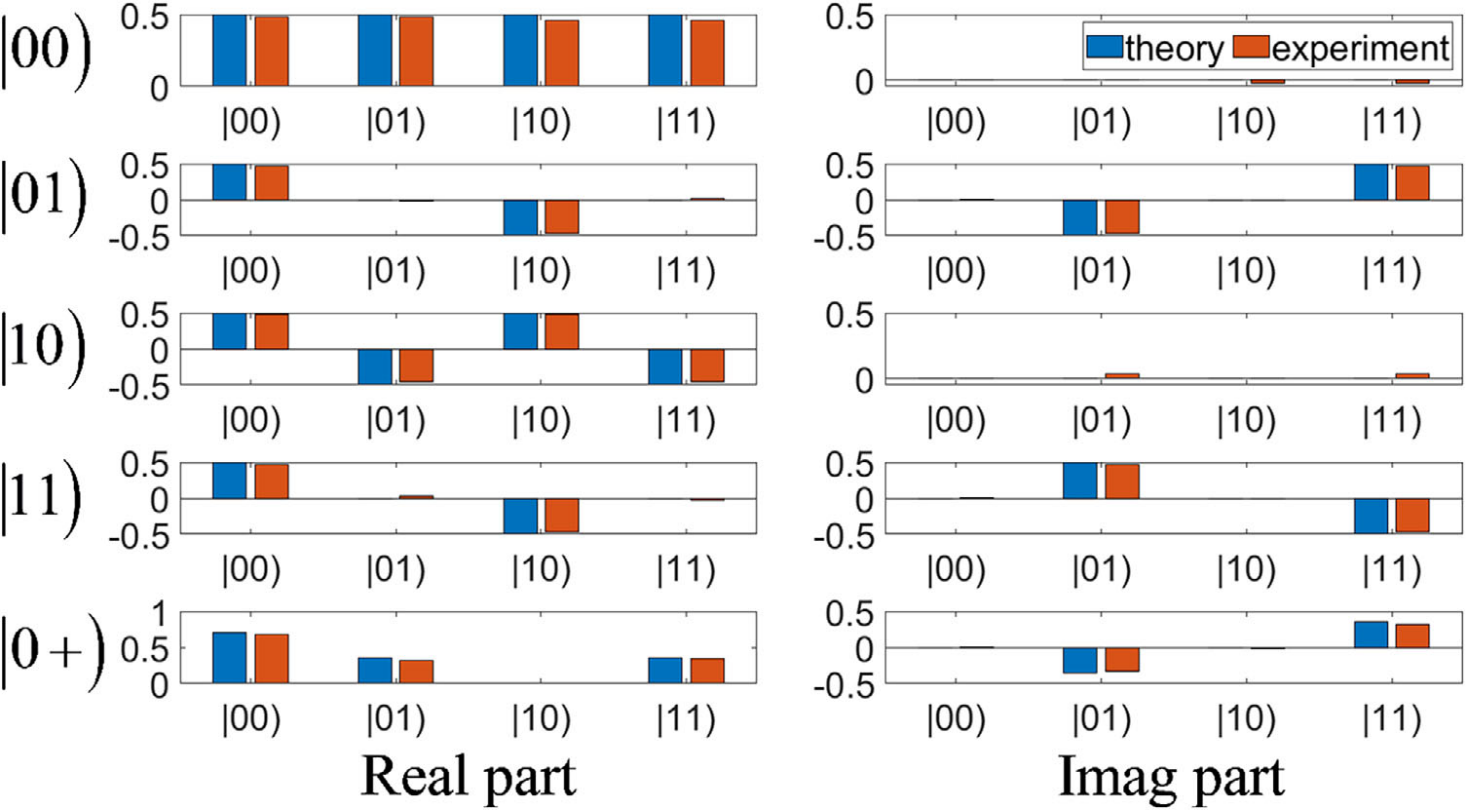
The theoretical and experimental results of the circuit simulation of the 2‐qubit QFT.

The theoretical and experimental results are provided in **Figure** **4**. From top to bottom, the panels correspond to the input states |00), |10), |01), |11) and |0 +). The left column displays the real parts of the results, while the right column displays the imaginary parts. The horizontal axis represents the states, while the vertical axis represents the amplitudes. The blue bar plots represent the theoretical results of QFT

(46) $$\left.\left|00\right.\right)\overset{\mathrm{QFT}}{\to }\frac{\left.\left|00\right.\right)+\left.\left|01\right.\right)+\left.\left|10\right.\right)+\left.\left|11\right.\right)}{2},$$

(47) $$\left.\left|01\right.\right)\overset{\mathrm{QFT}}{\to }\frac{\left.\left|00\right.\right)-i\left.\left|01\right.\right)-\left.\left|10\right.\right)+i\left.\left|11\right.\right)}{2},$$

(48) $$\left.\left|10\right.\right)\overset{\mathrm{QFT}}{\to }\frac{\left.\left|00\right.\right)-\left.\left|01\right.\right)+\left.\left|10\right.\right)-\left.\left|11\right.\right)}{2},$$

(49) $$\left.\left|11\right.\right)\overset{\mathrm{QFT}}{\to }\frac{\left.\left|00\right.\right)+i\left.\left|01\right.\right)-\left.\left|10\right.\right)-i\left.\left|11\right.\right)}{2},$$

(50) $$\left.\left|0+\right.\right)\overset{\mathrm{QFT}}{\to }\frac{\left.2\left|00\right.\right)+\left.\left(1-i\right)\left|01\right.\right)+\left.\left(1+i\right)\left|11\right.\right)}{2\sqrt{2}},$$

while the orange bar plots represent the experimental results, consistent with the theoretical results. Among these classical states, the mathematical form of Equation (50) corresponds to the quantum entangled state $\frac{2|00\rangle +(1-i)|01\rangle +(1+i)|11\rangle }{2\sqrt{2}}$. That is, our constructed classical correlation in circuits is a concept different from the quantum correlations that are generally believed to exist only in quantum systems, but their mathematical forms are the same. In fact, not only do they have the same mathematical form, but they can also exhibit similar physical properties.

It is well known that there are typically two approaches to characterize the quantum correlations of entangled states, one using violation of the Bell inequality and the other using entanglement entropy. Similarly, the correlation properties of our constructed classical correlated states in circuits can also be tested by two corresponding ways. We first discuss the circuit version of the violation of the Bell inequality. Its adaptation requires projective measurements on the state corresponding to the Bell‐like state along three directions θ_1_,θ_2_, and θ_3_, and substitute the measurements into the classical version of the Bell‐like inequality

(51) $$\left|P\left(\theta_{1},\theta_{2}\right)-P\left(\theta_{1},\theta_{3}\right)\right|\leq 1-P\left(\theta_{2},\theta_{3}\right)$$

where *P*(θ_1_,θ_2_) represents the correlated measurement outcome when the first cebit is measured along θ_1_ and the second qubit along θ_2_. In traditional classical systems, this inequality cannot be violated.

**Figure 5:**
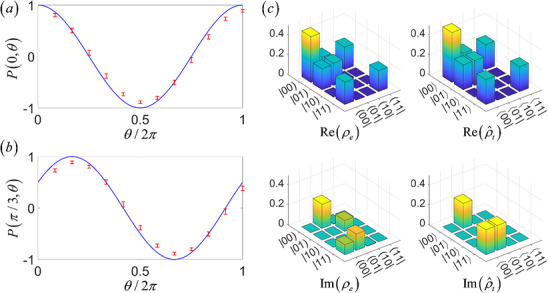
a,b) The variation of the correlated measurement outcomes *P*(θ′, θ) with θ under different parameter θ′. The blue line represents the theoretical results, while the red error bars represent the experimental results. c) The classical density matrix of the output classical state when the input classical state is |0 +) and the corresponding quantum density matrix. From left to right, the subfigures represent the real part of the classical density matrix of the experimental results, the real part of the quantum density matrix of the theoretical results, the imaginary part of the classical density matrix of the experimental results and the imaginary part of the quantum density matrix of the theoretical results.

In our circuit experiment, we first rotate the output state of the input state |0 +) into a classical state corresponding to the quantum Bell‐like state via two 1‐cebit gate described in Equation (39), and subsequently perform Bell‐like inequality measurements on the classical state corresponding to the quantum Bell state. **Figure** **5**a,b show the variation of *P*(θ′, θ) with θ under different parameter θ′. For parameters θ_1_ = 0, θ_2_ = π/3 and θ_3_ = 2π/3, we obtain *P*(θ_1_,θ_2_) = 0.51, *P*(θ_1_,θ_3_) = −0.39 and *P*(θ_2_,θ_3_) = 0.51. Consequently, the inequality in Equation (51) is violated. This means that although our constructed classical correlation in circuits is the concept different from the quantum correlations, it has a correlation relationship corresponding to the quantum correlations.

Now, we analyze the circuit version of the entanglement entropy. Its classical version requires the density matrix, which can be obtained through state tomography. The mathematical framework of state tomography remains unchanged, where projective measurements on the output state are performed on 4 states |0), |1), | +) and |*L*) for each cebit and the density matrix of the output state can then be resconstructed from these projective measurements results. However, these projective measurement operations require adaptation for classical implementation. In our circuit design, the correspondence to projective measurements can be divided into two step, the correspondence to projection and to the measurement. The 1‐cebit gate rotations implement the projection step, for example, the *U_H_* gate projects |0) to | +) and |1) to | −). And the operation that define the classical states by the voltage signals corresponds to the measurement step, for example, the amplitude φ′_00_ = 1/2 in Equation (46) indicates that the measured amplitude of the output voltage signal projected to |00) state is 1/2. Figure 5c presents the experimentally reconstructed classical density matrix ρ_*e*_ and the corresponding theoretical quantum density matrix ${\hat{\rho }}_{t}$. From left to right, the subfigures represent the real part of the classical density matrix of the experimental results, the real part of the quantum density matrix of the theoretical results, the imaginary part of the classical density matrix of the experimental results and the imaginary part of the quantum density matrix of the theoretical results.

Using the classical density matrix ρ_*e*_ of the output state, we compute the reduced classical density matrix ρ_*e*,1_ and calculate the classical correlation corresponding to the quantum entanglement entropy

(52) $$S\left(\rho_{e}\right)=-\mathrm{Tr}\left(\rho_{e,1}\ln\rho_{e,1}\right)=0.75=S\left({\hat{\rho }}_{t}\right)=>0.$$

These results also support the correspondence between the classical correlation among classical states of the electric circuits and the quantum entanglement in the quantum system.

**Figure 6:**
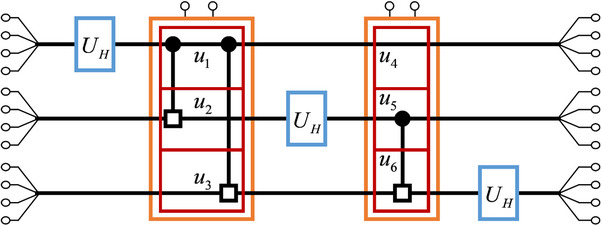
The schematic diagram of the 3‐cebit simulation circuit.

Based on the correspondence between classical correlation and quantum entanglement, our circuit design can be extended to scenarios with more cebits. We demonstrate the scalability of our circuit design through the two examples, the circuit simulation of 3‐qubit QFT and the theoretical simulation of 5‐qubit QFT. **Figure** **6** shows the schematic diagram of the 3‐cebit simulation circuit, which consists of 3 *U_H_* gates and 2*U_CR_* gate. The first *U_CR_* gate consists of 3 submodule called *u*_1_,*u*_2_ and *u*_3_, the second *U_CR_* gate consists of *u*_4_,*u*_5_ and *u*_6_. The submodules *u*_1_ and *u*_5_ act on the control cebit, containing 1 CR module. The submodules *u*_2_,*u*_3_ and *u*_6_ act on the target cebit, containing 2 CR modules. And the submodule *u*_4_ acts on the other cebit, containing 2 CR modules. The PCB layouts for these CR modules share a similar design with the PCB layouts shown in Figure 3c,d, but their specific configurations differ, as is shown in Method (B).

Theoretically, our 3‐cebit circuit is able to simulate the 3‐qubit QFT for arbitrary input states. For testing purposes, we arbitrarily selected a 3‐cebit state

(53) $$\begin{array}{c} \left.\left|\varphi \right.\right)=\left[\begin{array}{c} 0.38+0.38i\\ 0.61+0.59i \end{array}\right]⊗\left[\begin{array}{c} 0.53+0.31i\\ 0.66+0.43i \end{array}\right]⊗\left[\begin{array}{c} 0.24+0.65i\\ 0.61+0.38i \end{array}\right]\\ \;\;=\left[\begin{array}{c} -0.19+0.13i\\ -0.01+0.23i\\ -0.25+0.16i\\ -0.10+0.29i\\ -0.29+0.21i\\ -0.11+0.36i\\ -0.39+0.25i\\ -0.16+0.45i \end{array}\right]. \end{array}$$

It corresponds to following initial voltage configuration (voltage unit is V, and ω is set to 1kHz)

(54) $$\left[\begin{array}{c} V_{1,0,Re}\\ V_{1,0,Im}\\ V_{1,1,Re}\\ V_{1,1,Im} \end{array}\right]=\left[\begin{array}{c} 0.38\cos\left(\omega t\right)-0.38\sin\left(\omega t\right)\\ 0.38\cos\left(\omega t\right)+0.38\sin\left(\omega t\right)\\ 0.61\cos\left(\omega t\right)-0.59\sin\left(\omega t\right)\\ 0.59\cos\left(\omega t\right)+0.61\sin\left(\omega t\right) \end{array}\right],$$

(55) $$\left[\begin{array}{c} V_{2,0,Re}\\ V_{2,0,Im}\\ V_{2,1,Re}\\ V_{2,1,Im} \end{array}\right]=\left[\begin{array}{c} 0.53\cos\left(\omega t\right)-0.31\sin\left(\omega t\right)\\ 0.31\cos\left(\omega t\right)+0.53\sin\left(\omega t\right)\\ 0.66\cos\left(\omega t\right)-0.43\sin\left(\omega t\right)\\ 0.43\cos\left(\omega t\right)+0.66\sin\left(\omega t\right) \end{array}\right],$$

(56) $$\left[\begin{array}{c} V_{3,0,Re}\\ V_{3,0,Im}\\ V_{3,1,Re}\\ V_{3,1,Im} \end{array}\right]=\left[\begin{array}{c} 0.24\cos\left(\omega t\right)-0.65\sin\left(\omega t\right)\\ 0.65\cos\left(\omega t\right)+0.24\sin\left(\omega t\right)\\ 0.61\cos\left(\omega t\right)-0.38\sin\left(\omega t\right)\\ 0.38\cos\left(\omega t\right)+0.61\sin\left(\omega t\right) \end{array}\right].$$

**Figure 7:**
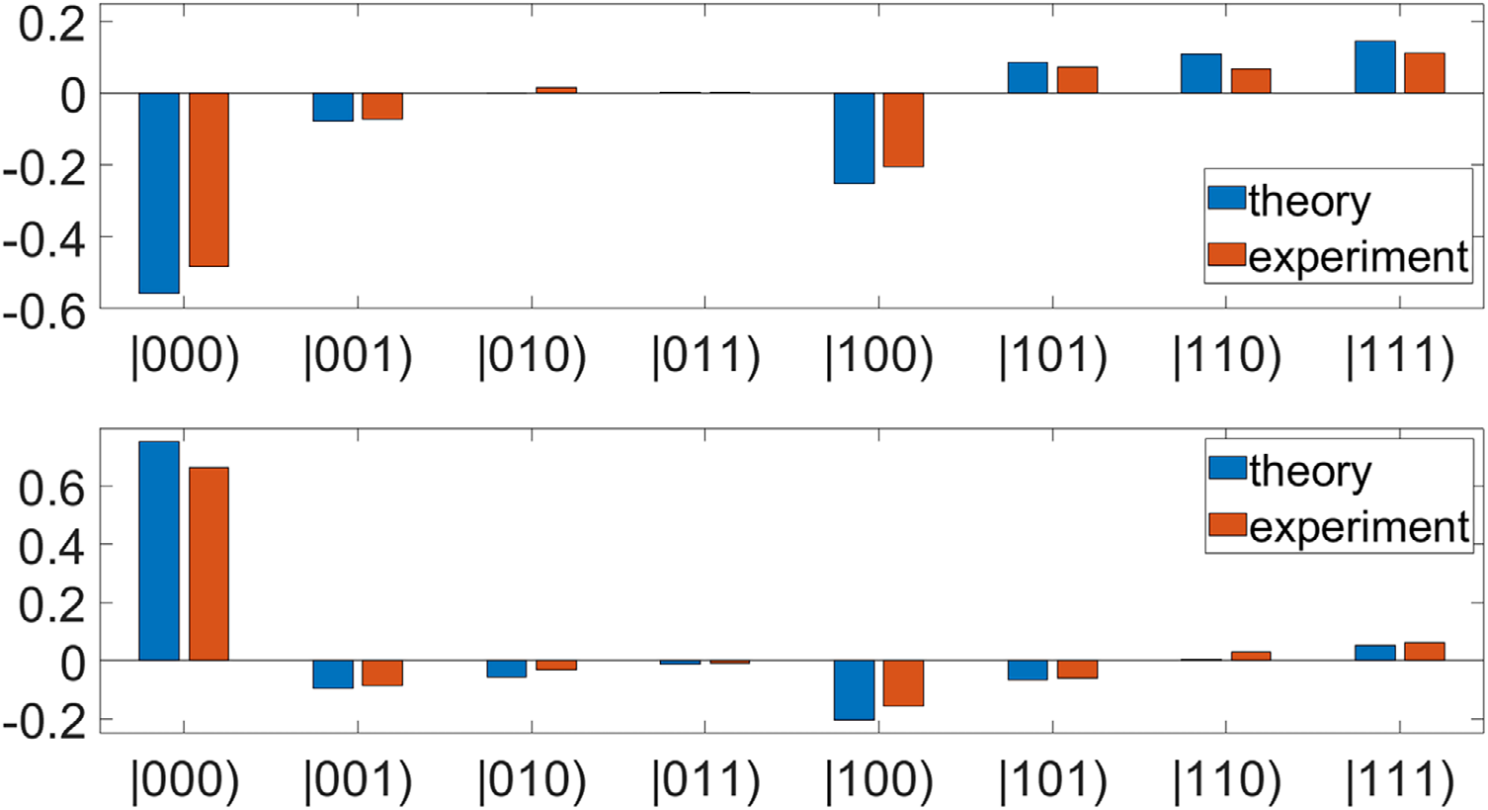
The theoretical and experimental results of the circuit simulation of the 3‐qubit QFT.

The theoretical and experimental results are provided in **Figure** **7**. The top subfigure displays the real parts of the results, while the bottom subfigure displays the imaginary parts of the results. The horizontal axis represents the states, with the vertical axis representing the amplitudes. The blue bar plots represent the theoretical results of QFT, and the orange bar plots represent the experimental results, consistent with the theoretical results.

**Figure 8:**
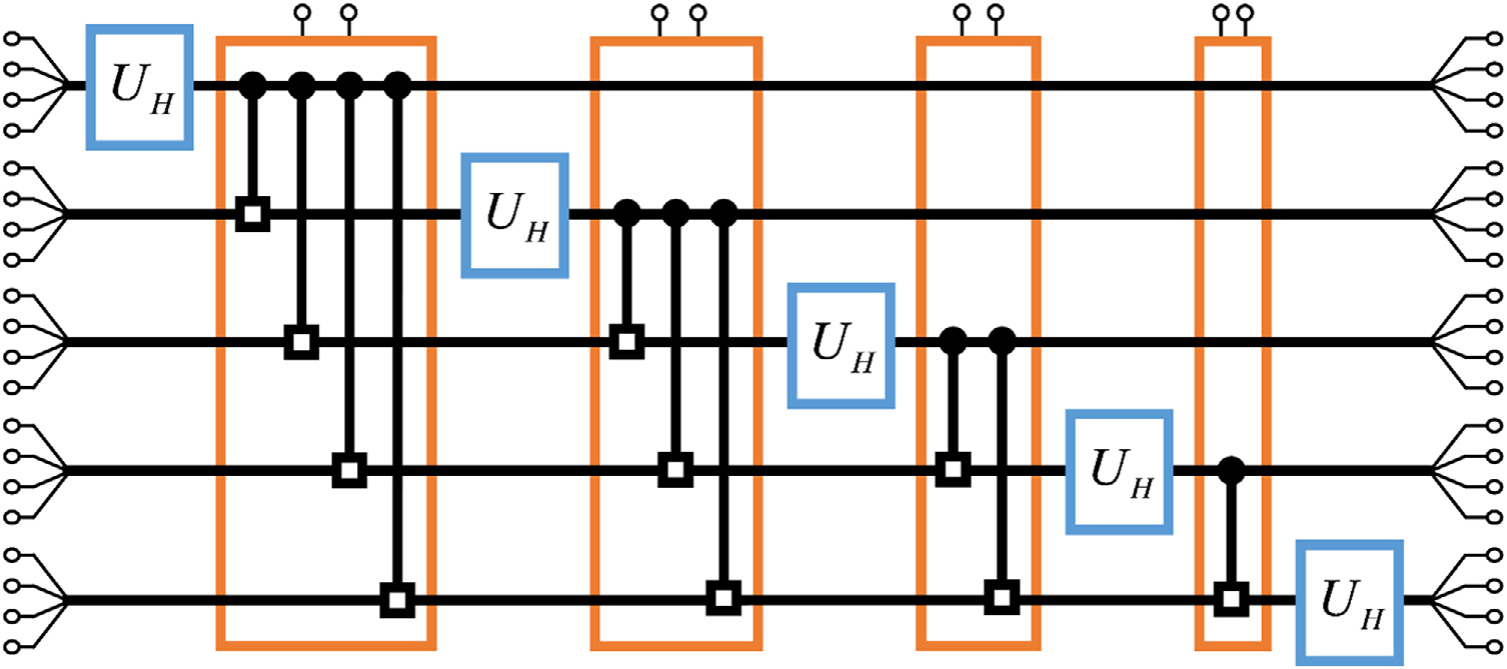
The schematic diagram of the 5‐cebit simulation circuit.

Similarly, **Figure** **8** displays the schematic diagram of the 5‐cebit simulation circuit. As discussed above, each circuit gate (blue and orange boxes) contains polynomial number of basic computing components. In this theoretical simulation, we arbitrarily select a 5‐cebit state

(57) $$\begin{array}{c} \left.\left|\varphi \right.\right)=\left[\begin{array}{c} 0.37+0.20i\\ 0.63+0.65i \end{array}\right]⊗\left[\begin{array}{c} 0.21+0.58i\\ 0.65+0.44i \end{array}\right]⊗\left[\begin{array}{c} 0.56+0.60i\\ 0.35+0.46i \end{array}\right]\\ \;\;\;⊗\left[\begin{array}{c} 0.59+0.03i\\ 0.46+0.66i \end{array}\right]⊗\left[\begin{array}{c} 0.37+0.26i\\ 0.67+0.59i \end{array}\right]. \end{array}$$

**Figure 9:**
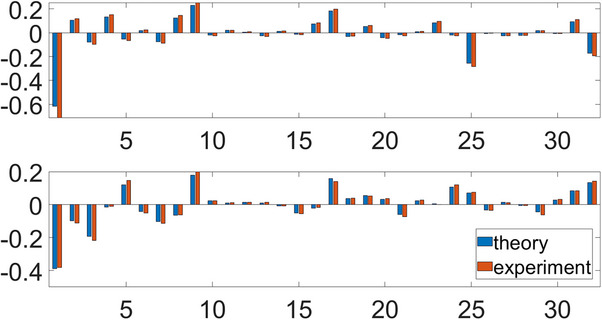
The theoretical results and the simulation results of the circuit simulation of the 5‐qubit QFT.

The theoretical results and the simulation results are provided in **Figure** **9**, with the accuracy of the circuit components configured to 1%. The top subfigure displays the real parts of the results, while the bottom subfigure displays the imaginary parts of the results. The horizontal axis represents the index of the states, with the vertical axis representing the amplitudes. The blue bar plots represent the theoretical results of QFT, and the orange bar plots represent the simulation results, consistent with the theoretical results.

## 4 Discussion and Conclusion

We have realized the circuit simulation of QFT, where the number of the basic computing components required in out circuit design matches that of quantum gates in the quantum circuit. The correctness of the 2‐cebit, 3‐cebit and 5‐cebit circuit simulation schemes has been validated. We summarize the principles underlying the speedup achieved by our simulation scheme as follows.

First, based on previous researches on classical correlation, we employ the correlated circuit wave systems as the experimental platform, which provides the foundation for demonstrating classical correlation in the systems. Second, we have successfully developed a novel definition of the classical states by the voltage signals, which mathematically corresponds to quantum entanglement, establishing the theoretical basis for our experiments. Within this scheme, a single computing component, processes all information in the waveform across one circuit channels, as one operation, enabling parallel computation over the entire dataset and thereby generating speedup. Notably, this parallel speedup relies on the existence of the novel classical correlations, thus traditional classical computing systems lacking such correlations cannot achieve this speedup. In traditional classical algorithms, significant time is consumed by operations such as permutation and selection within the input data itself. In contrast, the scheme presented in this work eliminates the need for any internal manipulation of the input data, requiring only global operations in the entire dataset.

Leveraging the mature, stable, and scalable nature of circuit platforms, our quantum‐inspired computing scheme based on circuit holds the potential to circumvent the challenges faced by quantum platforms and open a new way toward advanced information processing with high quality and efficiency. Furthermore, our work highlights the significant potential of analog computing in parallel speedup, which can be further optimized to realize practical speedup effects.

## 5 Experimental Section

### The Detailed Circuit Design of 1‐Cebit Gate (Adder Module)

**Figure 10:**
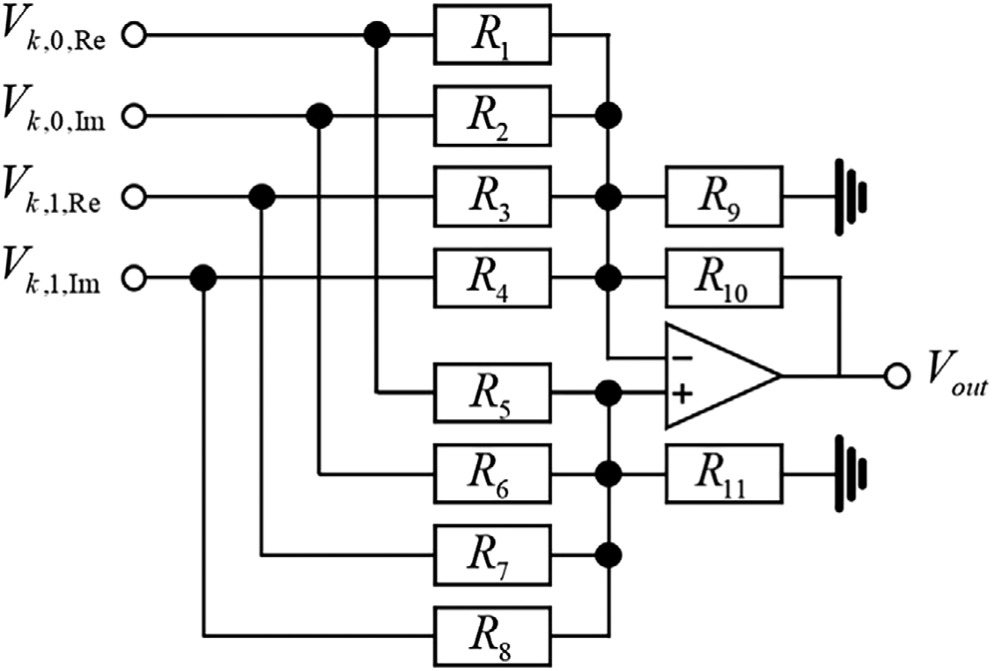
The circuit design of the arbitrarily 1‐cebit gate.

The 1‐cebit gate is an important basic gate in circuit design, and here the detailed design of the arbitrarily 1‐cebit gate is illustrated. It consists of 4 adder modules shown in **Figure** **10**. Each adder module contains 4 inputs, 1 operational amplifier, at most 11 resistors, and 1 output. The add module is referred to as “add circuit”, performing weighted addition (and subtraction) operations on the input voltage signals.

According to Kirchhoff's law,

(58) $$\begin{array}{cc} & 0=\frac{V_{-}-V_{k,0,Re}}{R_{1}}+\frac{V_{-}-V_{k,0,Im}}{R_{2}}+\frac{V_{-}-V_{k,1,Re}}{R_{3}}\\ & \;\;+\frac{V_{-}-V_{k,1,Im}}{R_{4}}+\frac{V_{-}}{R_{9}}+\frac{V_{-}-V_{out}}{R_{10}}+I_{-,in}, \end{array}$$

(59) $$\begin{array}{c} 0=\frac{V_{+}-V_{k,0,Re}}{R_{5}}+\frac{V_{+}-V_{k,0,Im}}{R_{6}}+\frac{V_{+}-V_{k,1,Re}}{R_{7}}\\ +\frac{V_{+}-V_{k,1,Im}}{R_{8}}+\frac{V_{+}}{R_{11}}+I_{+,in}. \end{array}$$

is observed. Subsequently, by combining the properties of operational amplifier that

(60) $$V_{-}=V_{+},I_{-,in}=I_{+,in}=0,$$

is obtained

(61) $$\begin{array}{cc} V_{out}= & \;\;\;\left(\frac{k}{R_{5}}-\frac{1}{R_{1}}\right)V_{k,0,Re}+\left(\frac{k}{R_{6}}-\frac{1}{R_{2}}\right)V_{k,0,Im}\\ & +\left(\frac{k}{R_{7}}-\frac{1}{R_{3}}\right)V_{k,1,Re}+\left(\frac{k}{R_{8}}-\frac{1}{R_{4}}\right)V_{k,1,Im}, \end{array}$$

where

(62) $$k=R_{10}\frac{\frac{1}{R_{1}}+\frac{1}{R_{2}}+\frac{1}{R_{3}}+\frac{1}{R_{4}}+\frac{1}{R_{9}}+\frac{1}{R_{10}}}{\frac{1}{R_{5}}+\frac{1}{R_{6}}+\frac{1}{R_{7}}+\frac{1}{R_{8}}+\frac{1}{R_{11}}}$$

By adjusting the resistance values, *V*_out_ can be configured as an arbitrary linear superposition of the inputs, thereby realizing any 1‐cebit gate. For example, in a 4‐input 4‐output circuit, the resistance values are adjusted for output $V_{1,0,Re}^{\prime }$ to (the resistance unit is kΩ)

(63) $$R_{5}=R_{7}=5\sqrt{2},R_{9}=\frac{10}{2\sqrt{2}-1},R_{10}=10.$$

Resistors not explicitly mentioned are disconnected from the circuit, and

(64) $$V_{1,0,Re}^{\prime }=\frac{V_{1,0,Re}+V_{1,1,Re}}{\sqrt{2}}$$

is observed. Similar configurations are applied to the remaining 3 outputs

(65) $$V_{1,0,Im}^{\prime }:R_{6}=R_{8}=5\sqrt{2},R_{9}=\frac{10}{2\sqrt{2}-1},R_{10}=10,$$

(66) $$V_{1,1,Re}^{\prime }:R_{3}=R_{5}=5\sqrt{2},R_{10}=R_{11}=10,\;\;\;\;$$

(67) $$V_{1,1,Im}^{\prime }:R_{4}=R_{6}=5\sqrt{2},R_{10}=R_{11}=10.\;\;\;\;$$

Resistors not explicitly mentioned are disconnected from the circuit, and the *U_H_* gate is obtained

(68) $$\left[\begin{array}{c} {V^{\prime }}_{1,0,Re}\\ {V^{\prime }}_{1,0,Im}\\ {V^{\prime }}_{1,1,Re}\\ {V^{\prime }}_{1,1,Im} \end{array}\right]=\frac{1}{\sqrt{2}}\left[\begin{array}{c} V_{1,0,Re}+V_{1,1,Re}\\ V_{1,0,Im}+V_{1,1,Im}\\ V_{1,0,Re}-V_{1,1,Re}\\ V_{1,0,Im}-V_{1,1,Im} \end{array}\right]$$

In the experiment circuit, specific resistance values can be achieved through rounding and series‐parallel combinations of resistors.

Furthermore, this adder module can be extended to contain any number of input signals, with the output equaling the weighted sum of all inputs. Since the underlying principle remains consistent, further elaboration is omitted here.

In the last, the detailed calculation for the 1‐cebit gate *U_H_*, and the arbitrary 1‐cebit gate *U_R_*, in *n*‐cebit system are discussed. The *U_H_* gate on the *k*_0_‐thcebit transforms the voltage signals into

(69) $$\left\{\begin{array}{c} \left[\begin{array}{c} {V^{\prime }}_{k,0,Re}\\ {V^{\prime }}_{k,0,Im}\\ {V^{\prime }}_{k,1,Re}\\ {V^{\prime }}_{k,1,Im} \end{array}\right]=\left[\begin{array}{c} a_{1}V_{k,0,Re}-b_{1}V_{k,0,Im}+a_{2}V_{k,1,Re}-b_{2}V_{k,1,Im}\\ b_{1}V_{k,0,Re}+a_{1}V_{k,0,Im}+b_{2}V_{k,1,Re}+a_{2}V_{k,1,Im}\\ a_{3}V_{k,0,Re}-b_{3}V_{k,0,Im}+a_{4}V_{k,1,Re}-b_{4}V_{k,1,Im}\\ b_{3}V_{k,0,Re}+a_{3}V_{k,0,Im}+b_{4}V_{k,1,Re}+a_{4}V_{k,1,Im} \end{array}\right],\;k=k_{0},\\ \left[\begin{array}{c} {V^{\prime }}_{k,0,Re}\\ {V^{\prime }}_{k,0,Im}\\ {V^{\prime }}_{k,1,Re}\\ {V^{\prime }}_{k,1,Im} \end{array}\right]=\left[\begin{array}{c} V_{k,0,Re}\\ V_{k,0,Im}\\ V_{k,1,Re}\\ V_{k,1,Im} \end{array}\right],\;\;\;\;k\neq k_{0}. \end{array}\right.$$

where *a*_1_ to *a*_4_ and *b*_1_ to *b*_4_ are adjustable circuit parameter. This expression is the result of extending Equation (6) to the *n*‐cebit system. Using the *n*‐cebit definition of the classical states in Equation (22), is observe

(70) $$\begin{array}{c} {\varphi^{\prime }}_{s_{k_{0}}=0}=\frac{1}{T}\int_{0}^{T}dt\sum_{j=1}^{W}e^{-ij\omega t}\left(\prod_{l\neq k}^{n}*\left({V^{\prime }}_{k,s_{k},Re}+i{V^{\prime }}_{k,s_{k},Im}\right)\right)\\ \;\;\;\;\;*\left({V^{\prime }}_{k_{0},0,Re}+i{V^{\prime }}_{k_{0},0,Im}\right)\\ \;\;\;\;=\frac{1}{T}\int_{0}^{T}dt\sum_{j=1}^{W}e^{-ij\omega t}\left(\prod_{l\neq k}^{n}*\left(V_{k,s_{k},Re}+iV_{k,s_{k},Im}\right)\right)\\ \;\;\;\;\;*\left(\left(a_{1}+ib_{1}\right)V_{k_{0},0,Re}+i\left(a_{1}+ib_{1}\right)V_{k_{0},0,Im}\right)\\ \;\;\;\;\;+\frac{1}{T}\int_{0}^{T}dt\sum_{j=1}^{W}e^{-ij\omega t}\left(\prod_{l\neq k}^{n}*\left(V_{k,s_{k},Re}+iV_{k,s_{k},Im}\right)\right)\\ \;\;\;\;\;\;*\left(\left(a_{2}+ib_{2}\right)V_{k_{0},1,Re}+i\left(a_{2}+ib_{2}\right)V_{k_{0},1,Im}\right)\\ \;\;\;=\left(a_{1}+ib_{1}\right)\varphi_{s_{k_{0}}=0}+\left(a_{2}+ib_{2}\right)\varphi_{s_{k_{0}}=1}, \end{array}$$

(71) $$\varphi_{s_{k_{0}}=1}^{\prime }=\left(a_{3}+ib_{3}\right)\varphi_{s_{k_{0}}=0}+\left(a_{4}+ib_{4}\right)\varphi_{s_{k_{0}}=1},$$

is observed, namely

(72) $$\left[\begin{array}{c} {\varphi^{\prime }}_{s_{k_{0}}=0}\\ {\varphi^{\prime }}_{s_{k_{0}}=1} \end{array}\right]=\left[\begin{array}{c} \left(a_{1}+ib_{1}\right)\varphi_{s_{k_{0}}=0}+\left(a_{2}+ib_{2}\right)\varphi_{s_{k_{0}}=1}\\ \left(a_{3}+ib_{3}\right)\varphi_{s_{k_{0}}=0}+\left(a_{4}+ib_{4}\right)\varphi_{s_{k_{0}}=1} \end{array}\right],$$

where $\varphi_{s_{k_{0}}=0}$ should be understood as selecting the components where the subscript $s_{k_{0}}=0$ from the entire set of $\varphi_{s_{1}s_{2}\text{…}s_{n}}$, and then forming a column vector composed of these selected components. And so are $\varphi_{s_{k_{0}}=1}$.

The computing details of the 4‐input 1‐input adder module are discussed. It can be simply extended to scenarios with additional inputs and outputs.

### The Detailed Circuit Design and the Output Calculation of CR Module

**Figure 11:**
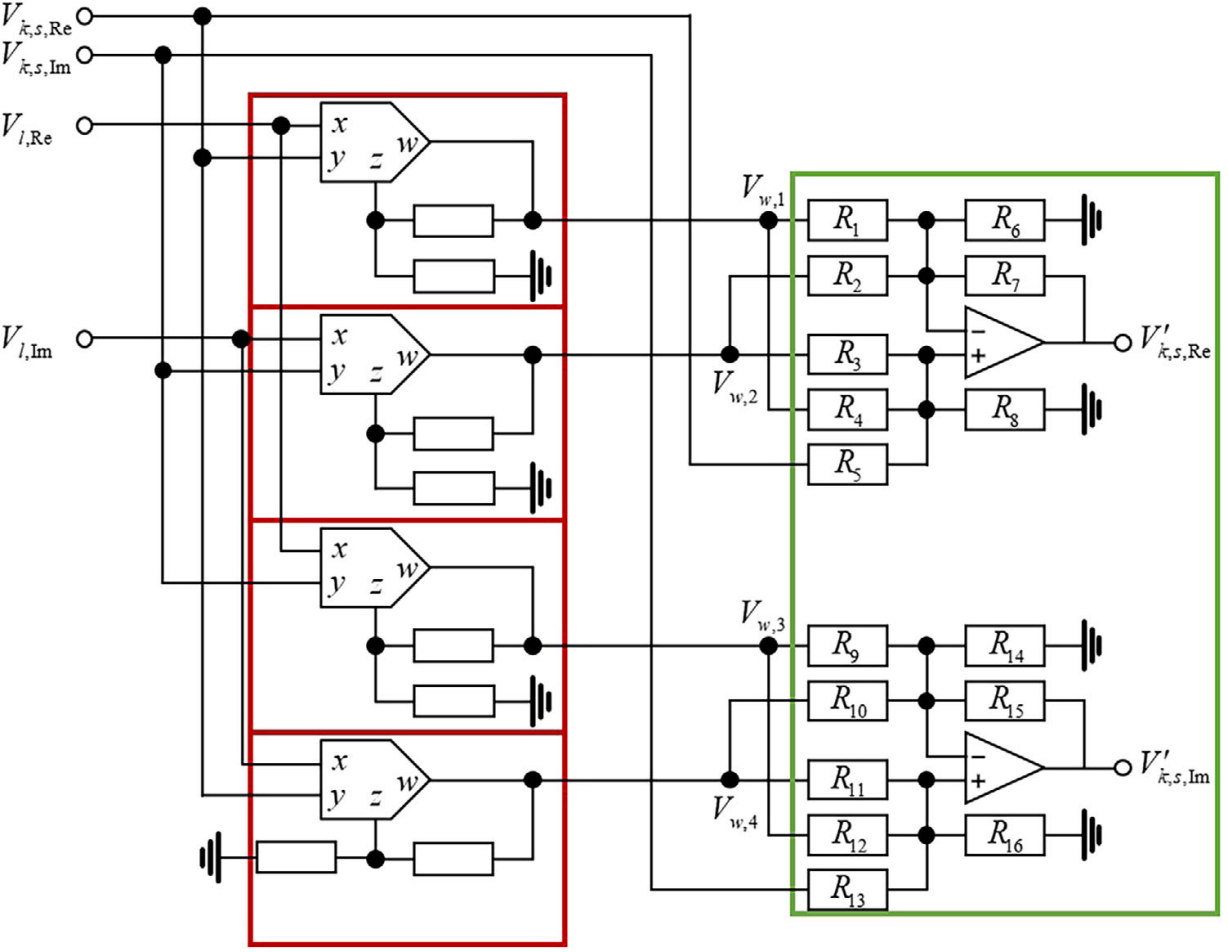
The circuit design of one certain CR module. It consists of analog multipliers (red box) and adder modules (green box).

The controlled phase gate $U_{CR_{m}}$ (and *U_CR_*) are another kind of important basic gate in the design of circuit. This kind of gate consists of CR modules, and here the detailed design of the CR modules is illustrated. **Figure** **11** shows one simple CR module, that contains 4 inputs, 4 analog multiplier, 2 operational amplifier, 24 resistors (8 in red box and 16 in green box) and 2 output.

Here *s* = 0 or 1, and the red box marks the fixed structure of the analog multiplier, with the output

(73) $$\left[\begin{array}{c} V_{w,1}\\ V_{w,2}\\ V_{w,3}\\ V_{w,4} \end{array}\right]=\left[\begin{array}{c} V_{l,Re}V_{k,s,Re}\\ V_{l,Im}V_{k,s,Im}\\ V_{l,Re}V_{k,s,Im}\\ V_{l,Im}V_{k,s,Re} \end{array}\right].$$

The red box marks the adder modules, with the output

(74) $$V_{k,s,Re}^{\prime }=V_{k,s,Re}+C_{1}V_{w,1}+C_{2}V_{w,2}$$

where *C*_1_ and *C*_2_ depend on the resistance values. If the resistance values are adjusted to

(75) $$R_{2}=R_{4}=R_{5}=R_{7}=R_{11}=R_{12}=R_{13}=R_{15}=10,R_{14}=5,$$

and resistors not explicitly mentioned are disconnected from the circuit, then

(76) $$\begin{array}{c} \left[\begin{array}{c} {V^{\prime }}_{k,s,Re}\\ {V^{\prime }}_{k,s,Im} \end{array}\right]=\left[\begin{array}{c} V_{k,s,Re}+V_{w,1}-V_{w,2}\\ V_{k,s,Re}+V_{w,3}+V_{w,4} \end{array}\right]\\ \;\;\;\;\;=\left[\begin{array}{c} V_{k,s,Re}+V_{l,Re}V_{k,s,Re}-V_{l,Im}V_{k,s,Im}\\ V_{k,s,Re}+V_{l,Re}V_{k,s,Im}+V_{l,Im}V_{k,s,Re} \end{array}\right]. \end{array}$$

is observed. Take *k* = 1 and *s* = 1, and $V_{1,1,Re}^{\prime }$ in the main text in Equation (14) is achieved. By adjusting the resistance values and the circuit connection, the voltage signals transformation in Equations (14) and (27) can be achieved. More generally, it can achieved

(77) $$\left\{\begin{array}{c} \begin{array}{ccc} {V^{\prime }}_{k,s,Re} & = & C_{1}V_{k,s,Re}+C_{2}V_{l,Re}V_{k,s,Re}+C_{3}V_{l,Re}V_{k,s,Im}\\ & & +C_{4}V_{l,Im}V_{k,s,Re}+C_{5}V_{l,Im}V_{k,s,Im}, \end{array}\\ \begin{array}{ccc} {V^{\prime }}_{k,s,Im} & = & C_{6}V_{k,s,Im}+C_{7}V_{l,Re}V_{k,s,Re}+C_{8}V_{l,Re}V_{k,s,Im}\\ & & +C_{9}V_{l,Im}V_{k,s,Re}+C_{10}V_{l,Im}V_{k,s,Im}, \end{array} \end{array}\right.$$

where the coefficients *C* depend on the resistance values in the adder module.

In the last, it is demonstrated that the output classical states of the *U_CR_* gate correspond to the results of quantum ${\hat{U}}_{CR}$ gate. In the *k*‐th cebit, suppose that $V_{k,s,Re}$ and $V_{k,s,Im}$ consists of sine waves and cosine waves with *W* different frequencies

(78) $$\left[\begin{array}{c} V_{k,s,Re}\\ V_{k,s,Im} \end{array}\right]=\sum_{w=1}^{W}\left[\begin{array}{c} A_{k,s,Re,w}\cos\left(w\omega t\right)-B_{k,s,Im,w}\sin\left(w\omega t\right)\\ A_{k,s,Re,w}\sin\left(w\omega t\right)+B_{k,s,Im,w}\cos\left(w\omega t\right) \end{array}\right],$$

or equivalently expressed as

(79) $$V_{k,s,Re}+iV_{k,s,Im}=\sum_{w=1}^{W}\left(A_{k,s,Re,w}+iB_{k,s,Im,w}\right)e^{iw\omega t}.$$

And the fixed inputs in the multiplier module are set as

(80) $$V_{l,Re}=\cos\left(W\omega t\right),V_{l,Im}=\sin\left(W\omega t\right).$$

The output voltage signals of the *k*_1_‐th cebit are

(81) $$\begin{array}{ccc} \left[\begin{array}{c} {V^{\prime }}_{k_{1},0,Re}\\ {V^{\prime }}_{k_{1},0,Im}\\ {V^{\prime }}_{k_{1},1,Re}\\ {V^{\prime }}_{k_{1},1,Im} \end{array}\right] & = & \left[\begin{array}{c} V_{k_{1},0,Re}\\ V_{k_{1},0,Im}\\ V_{l,Re}V_{k_{1},1,Re}-V_{l,Im}V_{k_{1},1,Im}\\ V_{l,Re}V_{k_{1},1,Im}+V_{l,Im}V_{k_{1},1,Re} \end{array}\right]\\ & = & \sum_{w=1}^{W}\left[\begin{array}{c} A_{k_{1},0,Re,w}\cos\left(w\omega t\right)-B_{k_{1},0,Im,w}\sin\left(w\omega t\right)\\ A_{k_{1},0,Re,w}\sin\left(w\omega t\right)+B_{k_{1},0,Im,w}\cos\left(w\omega t\right)\\ A_{k_{1},1,Re,w}\cos\left(\left(W+w\right)\omega t\right)-B_{k_{1},1,Im,w}\sin\left(\left(W+w\right)\omega t\right)\\ A_{k_{1},1,Re,w}\sin\left(\left(W+w\right)\omega t\right)+B_{k_{1},1,Im,w}\cos\left(\left(W+w\right)\omega t\right) \end{array}\right] \end{array}$$

or equivalently expressed as

(82) $$V_{k_{1},s,Re}^{\prime }+iV_{k_{1},s,Im}^{\prime }=\sum_{w=1}^{2W}\left({A^{\prime }}_{k_{1},s,Re,w}+i{B^{\prime }}_{k_{1},s,Im,w}\right)e^{iw\omega t},$$

where

(83) $$\begin{array}{c} \left\{\begin{array}{ccc} {A^{\prime }}_{k_{1},0,Re,w}=A_{k_{1},0,Re,w}, & {B^{\prime }}_{k_{1},0,Im,w}=B_{k_{1},0,Im,w}, & \left(0<w\leq W\right)\\ {A^{\prime }}_{k_{1},0,Re,w}=0, & {B^{\prime }}_{k_{1},0,Im,w}=0, & \left(W<w\leq 2W\right)\\ {A^{\prime }}_{k_{1},1,Re,w}=0, & {B^{\prime }}_{k_{1},1,Im,w}=0, & \left(0<w\leq W\right)\\ {A^{\prime }}_{k_{1},1,Re,w}=A_{k_{1},1,Re,w-W}, & {B^{\prime }}_{k_{1},1,Im,w}=B_{k_{1},1,Im,w-W}. & \left(W<w\leq 2W\right) \end{array}\right. \end{array}$$

Since the output signals share the same mathematical structure as the input signals, it can be directly utilized as the input signals for the subsequent circuit gate.

The output voltage signals of the *k*_0_‐th cebit and the *k*_2_‐thcebit are

(84) $$\left[\begin{array}{c} {V^{\prime }}_{k_{0},0,Re}\\ {V^{\prime }}_{k_{0},0,Im}\\ {V^{\prime }}_{k_{0},1,Re}\\ {V^{\prime }}_{k_{0},1,Im} \end{array}\right]=\left[\begin{array}{c} V_{k_{0},0,Re}+V_{l,Re}V_{k_{0},0,Re}-V_{l,Im}V_{k_{0},0,Im}\\ V_{k_{0},0,Im}+V_{l,Re}V_{k_{0},0,Im}+V_{l,Im}V_{k_{0},0,Re}\\ V_{k_{0},1,Re}+V_{l,Re}V_{k_{0},1,Re}-V_{l,Im}V_{k_{0},1,Im}\\ V_{k_{0},1,Im}+V_{l,Re}V_{k_{0},1,Im}+V_{l,Im}V_{k_{0},1,Re} \end{array}\right],$$

(85) $$\begin{array}{c} \left[\begin{array}{c} {V^{\prime }}_{k_{2},0,Re}\\ {V^{\prime }}_{k_{2},0,Im}\\ {V^{\prime }}_{k_{2},1,Re}\\ {V^{\prime }}_{k_{2},1,Im} \end{array}\right]=\left[\begin{array}{c} V_{k_{2},0,Re}+V_{l,Re}V_{k_{2},0,Re}-V_{l,Im}V_{k_{2},0,Im}\\ V_{k_{2},0,Im}+V_{l,Re}V_{k_{2},0,Im}+V_{l,Im}V_{k_{2},0,Re}\\ V_{k_{2},1,Re}+\cos\left(2\pi /2^{m}\right)F_{Re}-\sin\left(2\pi /2^{m}\right)F_{Im}\\ V_{k_{2},1,Im}+\sin\left(2\pi /2^{m}\right)F_{Re}+\cos\left(2\pi /2^{m}\right)F_{Im} \end{array}\right].\\ \left(F_{Re}=V_{l,Re}V_{k_{2},1,Re}-V_{l,Im}V_{k_{2},1,Im},F_{Im}=V_{l,Re}V_{k_{2},1,Im}+V_{l,Im}V_{k_{2},1,Re}\right) \end{array}$$

or equivalently expressed as

(86) $$\begin{array}{c} \left\{\begin{array}{ccc} {A^{\prime }}_{k_{0},0,Re,w}=A_{k_{0},0,Re,w}, & {B^{\prime }}_{k_{0},0,Im,w}=B_{k_{0},0,Im,w}, & \left(0<w\leq W\right)\\ {A^{\prime }}_{k_{0},0,Re,w}=A_{k_{0},0,Re,w}, & {B^{\prime }}_{k_{0},0,Im,w}=B_{k_{0},0,Im,w}, & \left(W<w\leq 2W\right)\\ {A^{\prime }}_{k_{0},1,Re,w}=A_{k_{0},1,Re,w-W}, & {B^{\prime }}_{k_{0},1,Im,w}=B_{k_{0},1,Im,w-W}, & \left(0<w\leq W\right)\\ {A^{\prime }}_{k_{0},1,Re,w}=A_{k_{0},1,Re,w-W}, & {B^{\prime }}_{k_{0},1,Im,w}=B_{k_{0},1,Im,w-W}, & \left(W<w\leq 2W\right) \end{array}\right. \end{array}$$

where

(87) $$\begin{array}{c} \left\{\begin{array}{ccc} {A^{\prime }}_{k_{0},0,Re,w}=A_{k_{0},0,Re,w}, & {B^{\prime }}_{k_{0},0,Im,w}=B_{k_{0},0,Im,w}, & \left(0<w\leq W\right)\\ {A^{\prime }}_{k_{0},0,Re,w}=A_{k_{0},0,Re,w}, & {B^{\prime }}_{k_{0},0,Im,w}=B_{k_{0},0,Im,w}, & \left(W<w\leq 2W\right)\\ {A^{\prime }}_{k_{0},1,Re,w}=A_{k_{0},1,Re,w-W}, & {B^{\prime }}_{k_{0},1,Im,w}=B_{k_{0},1,Im,w-W}, & \left(0<w\leq W\right)\\ {A^{\prime }}_{k_{0},1,Re,w}=A_{k_{0},1,Re,w-W}, & {B^{\prime }}_{k_{0},1,Im,w}=B_{k_{0},1,Im,w-W}, & \left(W<w\leq 2W\right) \end{array}\right. \end{array}$$

and

(88) $$\begin{array}{c} \left\{\begin{array}{ccc} {A^{\prime }}_{k_{2},0,Re,w}=A_{k_{2},0,Re,w}, & {B^{\prime }}_{k_{2},0,Im,w}=B_{k_{2},0,Im,w}, & \left(0<w\leq W\right)\\ {A^{\prime }}_{k_{2},0,Re,w}=A_{k_{2},0,Re,w}, & {B^{\prime }}_{k_{2},0,Im,w}=B_{k_{2},0,Im,w}, & \left(W<w\leq 2W\right)\\ {A^{\prime }}_{k_{2},1,Re,w}=A_{k_{2},1,Re,w-W}, & {B^{\prime }}_{k_{2},1,Im,w}=B_{k_{2},1,Im,w-W}, & \left(0<w\leq W\right)\\ \begin{array}{c} {A^{\prime }}_{k_{2},1,Re,w}\\ \;=e^{i2\pi /2^{m}}A_{k_{2},1,Re,w-W}, \end{array} & \begin{array}{c} {B^{\prime }}_{k_{2},1,Im,w}\\ \;=e^{i2\pi /2^{m}}B_{k_{2},1,Im,w-W}, \end{array} & \left(W<w\leq 2W\right) \end{array}\right. \end{array}$$

Since the output signals share the same mathematical structure as the input signals, it can be directly utilized as the input signals for the subsequent circuit gate. Using the *n*‐cebit definition of the classical states in Equation (22),

(89) $$\begin{array}{c} \begin{array}{cc} & {\varphi^{\prime }}_{s_{1}s_{2}\text{…}s_{n}}\\ & \;=\frac{1}{T}\int_{0}^{T}dt\sum_{j=1}^{W}e^{-ij\omega t}\prod_{k=1}^{n}*\left({V^{\prime }}_{k,s_{k},Re}+i{V^{\prime }}_{k,s_{k},Im}\right)\\ & \;=\frac{1}{T}\int_{0}^{T}dt\sum_{j=1}^{2W}e^{-ij\omega t}\prod_{k=1}^{n}*\left(\sum_{w=1}^{2W}\left({A^{\prime }}_{k,s_{k},Re,w}+i{B^{\prime }}_{k,s_{k},Im,w}\right)e^{iw\omega t}\right)\\ & \;=\frac{1}{T}\int_{0}^{T}dt\sum_{j=1}^{2W}e^{-ij\omega t}\prod_{k=1}^{n}*\left(\sum_{w=1}^{W}\left({A^{\prime }}_{k,s_{k},Re,w}+i{B^{\prime }}_{k,s_{k},Im,w}\right)e^{iw\omega t}\right)\\ & \;\;+\frac{1}{T}\int_{0}^{T}dt\sum_{j=1}^{2W}e^{-ij\omega t}\prod_{k=1}^{n}*\left(\sum_{w=W+1}^{2W}\left({A^{\prime }}_{k,s_{k},Re,w}+i{B^{\prime }}_{k,s_{k},Im,w}\right)e^{iw\omega t}\right). \end{array} \end{array}$$

is observed. Note that the convolution operation has the following property

(90) $$\begin{array}{ccc} C_{w_{1}}e^{iw_{1}\omega t}*C_{w_{2}}e^{iw_{2}\omega t} & = & C_{w_{1}}C_{w_{2}}\frac{1}{T}\int_{0}^{T}d\tau e^{iw_{1}\omega \tau }e^{iw_{2}\omega \left(t-\tau \right)}\\ & = & \delta_{w_{1},w_{2}}C_{w_{1}}C_{w_{2}}e^{iw_{1}\omega t}. \end{array}$$

When $s_{k_{1}}=0$,

(91) $$\begin{array}{c} \left\{\begin{array}{cc} {A^{\prime }}_{k_{1},0,Re,w}+i{B^{\prime }}_{k_{1},0,Im,w}=0, & \left(W<w\leq 2W\right)\\ {A^{\prime }}_{k_{0},s_{k_{0}},Re,w}+i{B^{\prime }}_{k_{0},s_{k_{0}},Im,w}=A_{k_{0},s_{k_{0}},Re,w}+iB_{k_{0},s_{k_{0}},Im,w}, & \left(0<w\leq W\right)\\ {A^{\prime }}_{k_{1},0,Re,w}+i{B^{\prime }}_{k_{1},0,Im,w}=A_{k_{1},0,Re,w}+iB_{k_{1},0,Im,w}, & \left(0<w\leq W\right)\\ {A^{\prime }}_{k_{2},s_{k_{2}},Re,w}+i{B^{\prime }}_{k_{2},s_{k_{2}},Im,w}=A_{k_{2},s_{k_{2}},Re,w}+iB_{k_{2},s_{k_{2}},Im,w}, & \left(0<w\leq W\right) \end{array}\right. \end{array}$$

is observed, such that for any values of $s_{k_{0}}$ and $s_{k_{2}}$,

(92) $$\begin{array}{ccc} \varphi_{s_{k_{0}},s_{k_{1}}=0,s_{k_{2}}}^{\prime } & = & \frac{1}{T}\int_{0}^{T}dt\sum_{j=1}^{2W}e^{-ij\omega t}\prod_{k=1}^{n}\\ & & *\left(\sum_{w=1}^{W}\left({A^{\prime }}_{k,s_{k},Re,w}+i{B^{\prime }}_{k,s_{k},Im,w}\right)e^{iw\omega t}\right)\\ & = & \varphi_{s_{k_{1}}=0,s_{k_{2}}} \end{array}$$

is observed. When $s_{k_{1}}=1$,

(93) $$\begin{array}{c} \left\{\begin{array}{cc} {A^{\prime }}_{k_{1},0,Re,w}+i{B^{\prime }}_{k_{1},0,Im,w}=0, & \left(0<w\leq W\right)\\ {A^{\prime }}_{k_{0},0,Re,w}+i{B^{\prime }}_{k_{0},0,Im,w}=A_{k_{0},0,Re,w-W}+iB_{k_{0},0,Im,w-W}, & \left(W<w\leq 2W\right)\\ {A^{\prime }}_{k_{1},0,Re,w}+i{B^{\prime }}_{k_{1},0,Im,w}=A_{k_{1},0,Re,w-W}+iB_{k_{1},0,Im,w-W}, & \left(W<w\leq 2W\right)\\ {A^{\prime }}_{k_{2},0,Re,w}+i{B^{\prime }}_{k_{2},0,Im,w}=e^{i2\pi /2^{m}} & \\ \;\left(A_{k_{2},0,Re,w-W}+iB_{k_{2},0,Im,w-W}\right), & \left(W<w\leq 2W\right) \end{array}\right. \end{array}$$

is observed, where *m* = *k*_2_ − *k*_1_ + 1, and

(94) $$\begin{array}{ccc} & & {\varphi^{\prime }}_{\text{…}s_{k_{1}}=1,s_{k_{1}+1},s_{k_{1}+2},\text{…}s_{n}}\\ & & \;=\frac{1}{T}\int_{0}^{T}dt\sum_{j=1}^{2M}e^{-ij\omega t}\prod_{k=1}^{n}*\left(\sum_{w=W+1}^{2W}\left({A^{\prime }}_{k,s_{k},Re,w}+i{B^{\prime }}_{k,s_{k},Im,w}\right)e^{iw\omega t}\right)\\ & & \;=\prod_{s_{k_{2}}=1}e^{i2\pi /2^{m}}\varphi_{\text{…}s_{k_{1}}=1,s_{k_{1}+1},s_{k_{1}+2},\text{…}s_{n}} \end{array}$$

is observed. In other words, under the condition $s_{k_{1}}=1$, each term of $s_{k_{2}}=1$ in the subscript of the classical state function φ introduces a phase shift of 2π/2^*m*^(*m* = *k*_2_ − *k*_1_ + 1) in φ′. While in the ${\hat{U}}_{CR}$ gate, each term of $s_{k_{2}}=1$ in the subscript of the quantum state ϕ corresponds to an ${\hat{U}}_{CR_{m}}$ gate, which also introduces a 2π/2^*m*^ phase shift in ϕ′. Therefore, both the classical *U_CR_* gate and the quantum ${\hat{U}}_{CR_{m}}$ gate introduce identical phase shifts by the same subscript terms in their respective state functions, resulting in equivalent operational outcomes. This confirms that the circuit *U_CR_* gate corresponds to the quantum ${\hat{U}}_{CR_{m}}$ gate.

## Conflict of Interest

The authors declare no conflict of interest.

## Supporting information



Supporting Information

## Data Availability

The data that support the findings of this study are available in the supplementary material of this article.
